# Aryl hydrocarbon receptor restrains tonic cytokine responses by inhibiting microbiota sensing in monocytes

**DOI:** 10.1172/JCI189937

**Published:** 2025-10-02

**Authors:** Adeline Cros, Alessandra Rigamonti, Alba de Juan, Alice Coillard, Mathilde Rieux-Laucat, Darawan Tabtim-On, Emeline Papillon, Christel Goudot, Alma-Martina Cepika, Romain Banchereau, Virginia Pascual, Marianne Burbage, Burkhard Becher, Elodie Segura

**Affiliations:** 1 Institut Curie, PSL Research University, INSERM, U932, Paris, France.; 2 Institut Necker Enfants Malades – INEM, Université Paris Cité, INSERM, U1151, Paris, France.; 3 Baylor Institute for Immunology Research, Dallas, Texas, USA.; 4 Institute of Experimental Immunology, University of Zurich, Zurich, Switzerland.

**Keywords:** Immunology, Inflammation, Cytokines, Homeostasis, Monocytes

## Abstract

Immune cells are constantly exposed to microbiota-derived compounds that can engage innate recognition receptors. How this constitutive stimulation is downmodulated to avoid systemic inflammation and autoimmunity is poorly understood. Here, we show that aryl hydrocarbon receptor (AhR) deficiency in monocytes unleashed spontaneous cytokine responses in vivo, driven by stimulator of interferon genes (STING)-mediated tonic sensing of microbiota. This effect was specific to monocytes, as mice deficient for AhR specifically in macrophages did not show any dysregulation of tonic cytokine responses. AhR inhibition also increased tonic cytokine production in human monocytes. Finally, in patients with systemic juvenile idiopathic arthritis, low AhR activity in monocytes correlated with elevated cytokine responses. Our findings reveal an essential role for AhR in monocytes in restraining tonic microbiota sensing and in maintaining immune homeostasis.

## Graphical abstract

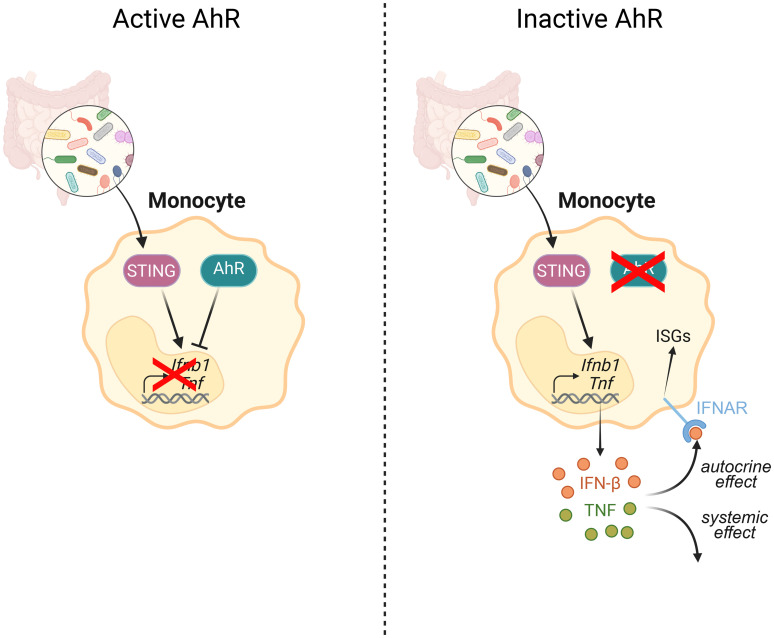

## Introduction

The organism is constantly exposed to microbiota-derived compounds that can engage immune defense receptors (1). This constitutive low-level stimulation, termed “tonic stimulation,” is essential for shaping the immune system but must be tightly controlled to prevent systemic inflammation and autoimmunity. How tonic sensing of commensal microbiota is counterbalanced in immune cells remains poorly understood.

Numerous studies using germ-free mice or microbiota depletion with antibiotics have evidenced the critical role of tonic microbiota sensing for regulating hematopoiesis, educating immune cells, and protecting against pathogen infections (1–3). Although mucosal barriers prevent microbiota penetration, microbiota-derived compounds can circulate systemically and be sensed by immune cells at distant sites, including bone marrow (4, 5) and spleen (6, 7). In particular, studies have shown the circulation of bacterial ligands for innate immune receptors including NOD-like receptors (4, 7, 8), TLRs (5, 9), and stimulator of interferon genes (STING) (10). Such ligands were shown to be disseminated via commensal bacteria–derived membrane vesicles (10).

Tonic microbiota sensing by immune cells induces the production of low levels of cytokines, in particular type I IFN (IFN-I). Tonic IFN-I signaling plays a critical role in licensing immune cells for efficient immune responses to infectious challenges, including mononuclear phagocytes (6, 11–13) and NK cells (14). DCs were shown to be the main source of constitutive IFN-I production, via the recognition of bacterial motifs at both mucosal and distant sites (6, 12, 15). Whether other immune cells, such as monocytes, equipped with similar recognition receptors participate in tonic IFN-I responses has remained unclear.

Tonic microbiota sensing can also induce TNF production by mononuclear phagocytes in the bone marrow (5), which participates in regulating hematopoiesis. Steady-state analysis of mice deficient for TNF or TNF receptor 1 revealed an essential role for constitutive TNF signaling in the establishment of normal lymphoid organ architecture (16, 17), in monocyte survival (18) and in intestinal homeostasis (19). Although microbiota sensing is a likely trigger for steady-state TNF production, this aspect was not investigated in these studies. Finally, tonic TNF signaling was also observed in cell culture and is critical for conditioning macrophages for optimal responses to acute microbial challenge (20).

While tonic cytokine responses play a major role in maintaining immune homeostasis, excessive cytokine production can lead to systemic inflammation and tissue damage (7, 21). In particular, elevated IFN-I signaling is associated with autoimmune pathologies (22). Numerous negative feedback mechanisms have been identified for inhibiting the recognition receptor signaling following microbial stimulation during infections (23). By contrast, the mechanisms restraining the magnitude of systemic immune responses during tonic sensing remain poorly understood (2). Activation of TLR signaling by microbiota-derived compounds is restrained by the ubiquitin-editing enzyme A20 (21) and the adaptor molecule TANK (24), both inhibiting NF-κB signaling and preventing uncontrolled cytokine production (including TNF and IL-6) and autoimmune disorders. Given the fundamental importance of the negative regulation of tonic microbiota sensing, additional mechanisms must exist, in particular for other sensing pathways.

Aryl hydrocarbon receptor (AhR) is a ligand-activated transcription factor expressed in multiple immune cells. Physiological agonists for AhR include tryptophan metabolites generated through food processing and microbiota metabolism (25). In the context of viral infections, AhR signaling was shown to downmodulate IFN-I responses via the induction of TIPARP, which inhibits TANK-binding kinase 1 (TBK1) downstream of RIG-I activation (26), or by inhibiting NF-κB signaling (27). Indeed, AhR activation in vitro decreases NF-κB signaling by inducing the negative regulator SOCS2 (28, 29). AhR signaling was also found in vitro to promote the ubiquitin-dependent proteasome degradation of the NF-κB subunit RelA (30) and of STING (31). Whether AhR plays a role in tonic immune responses is unknown.

By analyzing the role of AhR in mouse monocytes in the steady-state condition, we discovered that AhR deficiency in monocytes unleashed microbiota-driven cytokine responses. We also found that AhR signaling was essential in human monocytes to limit tonic cytokine production. These results shed light on the negative control of constitutive microbiota sensing and identify a key role for physiological activation of AhR in monocytes in the maintenance of immune homeostasis.

## Results

### Lack of AhR expression in monocytes causes dysregulation of tonic cytokine responses in vivo in mice.

**Figure 1:**
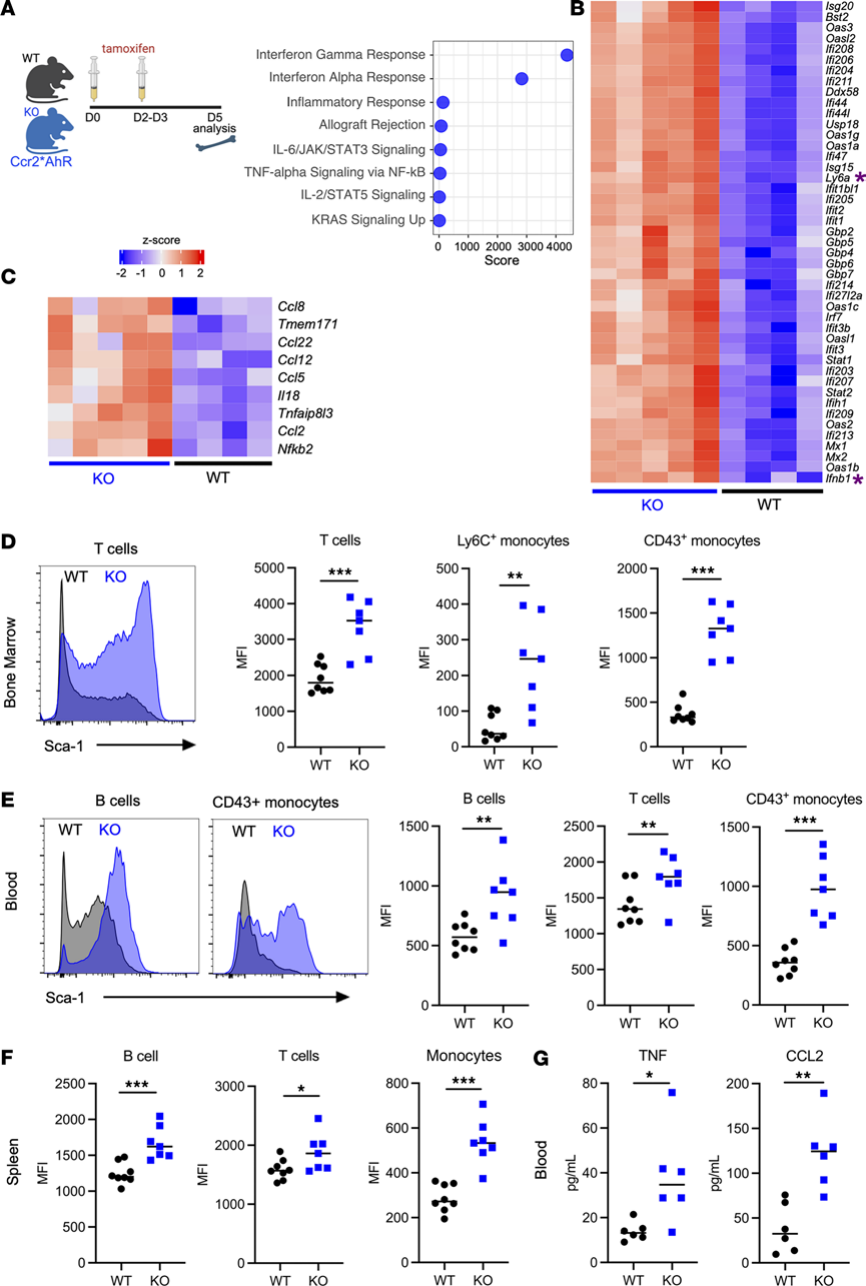
Deletion of AhR in monocytes by targeting *Ccr2*-expressing cells causes dysregulation of tonic cytokine responses in vivo. Ccr2*AhR^Δ^ mice (KO) or WT AhR^fl/fl^ littermates were treated with tamoxifen. (**A**–**C**) Monocytes from the bone marrow were purified and then subjected to RNA-Seq analysis (*n* = 4–5 biological replicates). (**A**) Enrichment analysis for Gene Ontology (GO) biological pathways using genes differentially upregulated in KO monocytes. D0, day zero. (**B**) Scaled expression of IFN-stimulated genes. Genes of special interest are highlighted by an asterisk. (**C**) Scaled expression of TNF-related genes. (**D**–**F**) Sca-1 expression in immune cell types of the indicated tissues from Ccr2*AhR^Δ^ mice or WT AhR^fl/fl^ littermates. Representative flow cytometric results are shown. For the MFI of Sca-1, the median is shown (*n* = 7–8 in 2 independent experiments). Significance was determined by Mann-Whitney *U* test. (**G**) Plasma concentration of the indicated cytokines in Ccr2*AhR^Δ^ mice (KO) and WT AhR^fl/fl^ littermates. The median is shown (*n* = 6 in 2 independent experiments). Significance was determined by Mann-Whitney *U* test. For all panels, *P < 0.05, **P < 0.01, and ***P < 0.001. An absence of asterisks indicates nonsignificance.

To address the role of AhR in monocytes, we generated mice in which *AhR* deletion was targeted to classical monocytes, by crossing *AhR^fl/fl^* mice with *Ccr2*-CreER^T2^ mice (32). This construction was reported to allow efficient deletion in Ly6C^+^ monocytes upon tamoxifen treatment, with marginal targeting of DC progenitors and their progeny (32, 33). We found that *AhR* expression in Ly6C^+^ monocytes was substantially reduced compared with expression in WT (Cre^–^) littermates in deficient mice harboring homozygous *Ccr2-*Cre alleles, but not heterozygous alleles (Supplemental Figure 1A; supplemental material available online with this article; https://doi.org/10.1172/JCI189937DS1). Therefore, in the rest of the study we used *Ccr2*-CreER^+/+^ *AhR^fl/fl^* mice as the deficient mice (Ccr2*AhR^Δ^). In addition, we verified that T cells and NK cells were not targeted for *AhR* deletion in these mice (Supplemental Figure 1B). To evaluate whether Ccr2*AhR^Δ^ mice had a defect in monocyte generation or maintenance, we assessed monocyte numbers in the bone marrow (Supplemental Figure 1, C and D). We found no significant difference in the number of Ly6C^+^ or Ly6C^–^CD43^+^ monocytes between Ccr2*AhR^Δ^ mice and their WT littermates (Supplemental Figure 1D). To address the effect on monocytes of the lack of AhR expression in vivo, we performed RNA-Seq analysis of AhR-deficient or WT bone marrow monocytes at steady state. We found only 35 genes significantly downregulated in AhR-deficient monocytes compared with WT monocytes (Supplemental Figure 1E and Supplemental Table 1). Among these genes, we did not identify any canonical AhR target genes, consistent with the absence of AhR stimulation by an exogenous agonist in this setting. By contrast, 263 genes were upregulated in AhR-deficient monocytes compared with WT (Supplemental Table 1). Pathway enrichment analysis indicated increased IFN responses and cytokine signaling, in particular that of TNF (Figure 1A). Indeed, *Ifnb1* and multiple IFN-stimulated genes (ISGs) (Figure 1B) as well as TNF-related genes, including *Ccl2* (Figure 1C), were upregulated in AhR-deficient monocytes. To validate these results at the functional level, we used as a readout the expression of Sca-1 (encoded by *Ly6a*), a very sensitive ISG detectable by flow cytometry (34). To address whether IFN-β secretion by monocytes could induce ISG expression in a paracrine fashion, we also included other immune cells in our analysis. Sca-1 expression was significantly increased in bone marrow monocytes and T cells of Ccr2*AhR^Δ^ mice (Figure 1D and Supplemental Figure 2A). To exclude the possibility that this phenomenon was caused by Cre expression alone, we assessed Sca-1 expression in bone marrow cells of mice with homozygous *Ccr2-*Cre alleles, but without any floxed *AhR* allele. *Ccr2*-CreER^+/+^ mice showed no marked upregulation of Sca-1 in CD43^+^ monocytes or in T cells (Supplemental Figure 2B), confirming that increased ISG expression was specific to mice with *AhR* deletion. Collectively, these results suggest that bone marrow AhR-deficient monocytes spontaneously secreted increased amounts of TNF and IFN-β, thereby activating cytokine responses in the steady-state context.

We noticed that Ccr2*AhR^Δ^ mice displayed hypomorphic expression of *Ccr2*, an effect that was dependent on the homozygous expression of *Ccr2-*Cre alleles but independent of *AhR* deletion (Supplemental Figure 2C). Because CCR2 is known to be critical for monocyte egress from the bone marrow (35, 36), we took advantage of this phenomenon to address whether *AhR* deletion in bone marrow monocytes was sufficient to induce systemic effects. To confirm that Ccr2*AhR^Δ^ mice had a defect in monocyte circulation, we examined peripheral monocytes in the blood (Supplemental Figure 2, D and E) and spleen (Supplemental Figure 2, F and G). Both Ly6C^+^ and CD43^+^ monocytes were significantly reduced in the blood of Ccr2*AhR^Δ^ mice, while blood neutrophil numbers remained unaffected (Supplemental Figure 2E). Monocyte numbers in the spleen were also decreased in Ccr2*AhR^Δ^ mice (Supplemental Figure 2G), confirming that Ccr2*AhR^Δ^ mice had defective monocyte egress from the bone marrow. We then analyzed Sca-1 expression in blood and spleen. In Ccr2*AhR^Δ^ mice, Sca-1 was upregulated in blood B cells, CD43^+^ monocytes, and T cells (Figure 1E) and in splenic B cells, monocytes, and T cells (Figure 1F and Supplemental Figure 2H) compared with WT mice. Finally, we found that TNF and CCL2 concentrations were increased in the plasma of Ccr2*AhR^Δ^ mice compared with WT littermates (Figure 1G). Collectively, these results indicate that a lack of AhR in monocytes unleashes spontaneous TNF and IFN-β production and increases tonic cytokine responses at a systemic level.

### AhR is required to regulate tonic cytokine responses in monocytes but not in macrophages.

**Figure 2:**
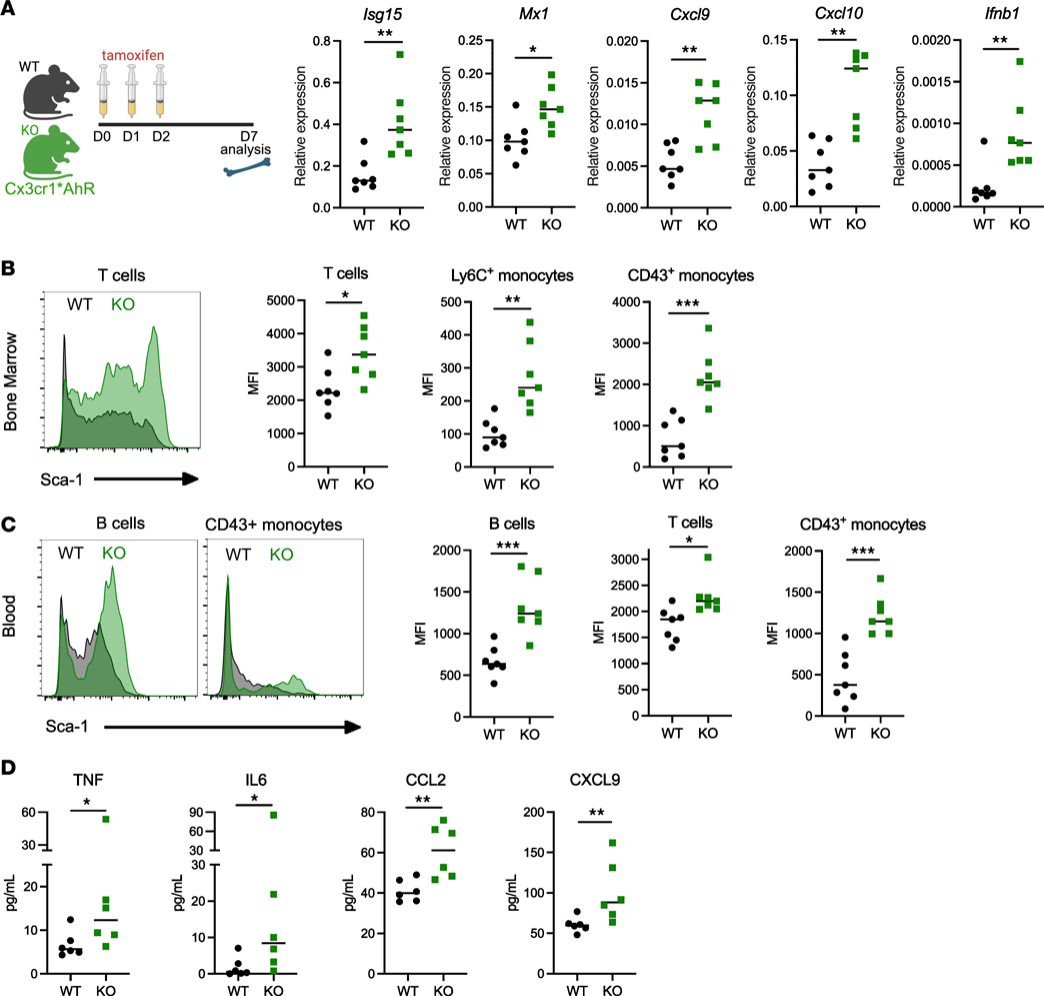
Deletion of AhR in monocytes by targeting *Cx3cr*1-expressing cells causes dysregulation of tonic cytokine responses in vivo. Cx3cr1*AhR^Δ^ mice (KO) and WT AhR^fl/fl^ littermates were treated with tamoxifen. (**A**) Monocytes from the bone marrow were purified. Expression of the indicated genes was assessed by reverse transcription quantitative pCR (RT-qPCR). The median is shown (*n* = 7 in 2 independent experiments). Schematic was created with BioRender. (**B** and **C**) Sca-1 expression in immune cell types of the indicated tissues from Cx3cr1*AhR^Δ^ mice or WT AhR^fl/fl^ littermates. Representative flow cytometric results are shown. For the MFI of Sca-1, the median is shown (*n* = 7 in 2 independent experiments). (**D**) Plasma concentration of the indicated cytokines in Cx3cr1*AhR^Δ^ mice (KO) and WT AhR^fl/fl^ littermates. The median is shown (*n* = 6 in 2 independent experiments). Significance was determined by Mann-Whitney *U* test. For all panels, *P < 0.05, **P < 0.01, and ***P < 0.001. An absence of asterisks indicates nonsignificance.

To further explore the role of AhR in the control of tonic cytokine responses, we generated another model for AhR-deficient monocytes by crossing *AhR^fl/fl^* mice with *Cx3cr1*-CreERT mice. *AhR* expression was significantly decreased in bone marrow Ly6C^+^ monocytes of Cx3cr1-Cre^+/–^*AhR*^fl/fl^* mice (Cx3cr1*AhR^Δ^) upon tamoxifen treatment (Supplemental Figure 3A). Peripheral monocyte numbers were unaffected, as evidenced in blood (Supplemental Figure 3B) and spleen (Supplemental Figure 3C). Bone marrow monocytes from Cx3cr1*AhR^Δ^ mice displayed spontaneous expression of *Ifnb1* and of ISGs (Figure 2A). In addition, Sca-1 expression was substantially elevated on bone marrow T cells and monocytes (Figure 2B and Supplemental Figure 3D) and in blood B cells, monocytes, and T cells (Figure 2C and Supplemental Figure 3D), indicating increased IFN-I responses at a systemic level, similar to the phenotype of Ccr2*AhR^Δ^ mice. Finally, we assessed cytokine levels in plasma. Circulating TNF, CCL2, CXCL9, and IL-6 levels were markedly elevated in Cx3cr1*AhR^Δ^ mice compared with levels in WT littermates (Figure 2D). Although these molecules may be produced by multiple cell types, these observations are consistent with a general dysregulation of steady-state cytokine production in Cx3cr1*AhR^Δ^ mice. Collectively, these results confirm that a lack of AhR in monocytes increased tonic cytokine responses.

Sexual dimorphism has been shown for IFN-I responses in mice and humans, in particular for mononuclear phagocytes (37–39). Because all of the above experiments were performed in female mice, we then addressed whether the effect of AhR deficiency on tonic cytokine responses in monocytes was sex biased. We compared female and male Cx3cr1*AhR^Δ^ mice and WT littermates for gene expression in bone marrow monocytes (Supplemental Figure 3E) and Sca-1 expression in bone marrow cells (Supplemental Figure 3F). Expression of *Isg15*, *Ifnb1*, and *Ccl5* was significantly increased in deficient monocytes to a similar extent in females and males. Sca-1 expression was also substantially increased in bone marrow monocytes and T cells in both male and female deficient mice. We conclude that AhR regulates tonic cytokine responses in monocytes similarly in female and male animals.

**Figure 3:**
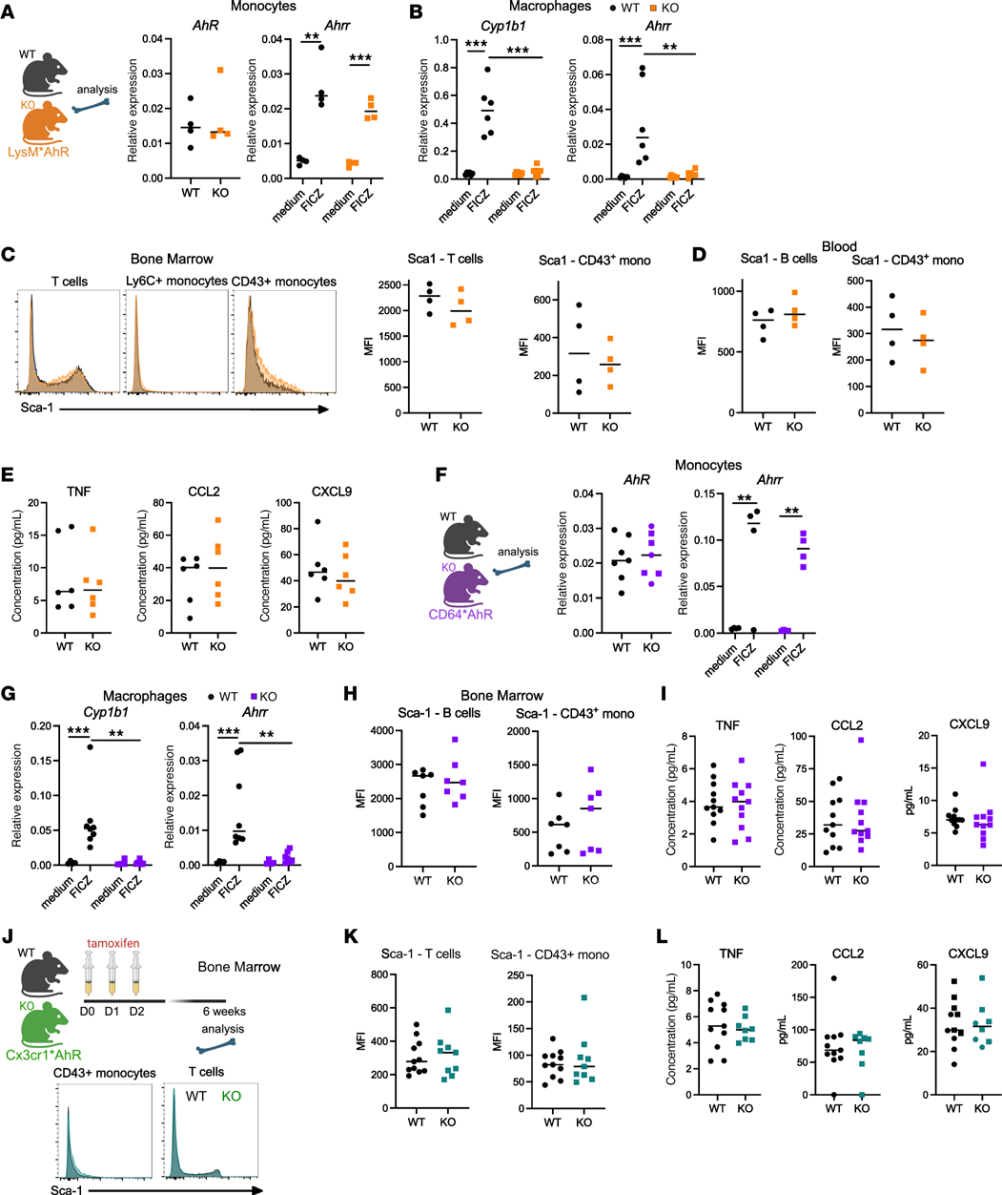
Tonic cytokine responses are unaffected in mice deficient for AhR in macrophages. (**A**–**E**) Lysm*AhR^Δ^ mice (KO) and WT AhR^fl/fl^ littermates were used. (**A**) *AhR* expression was assessed ex vivo on bone marrow monocytes, and *Ahrr* expression was assessed on monocytes cultured overnight with or without the AhR agonist FICZ. Gene expression was measured by RT-qPCR. The median is shown (*n* = 4 in 2 independent experiments). Mann-Whitney *U* test for *Ahr*, 2-way ANOVA for *Ahrr*. (**B**) Peritoneal macrophages were cultured overnight with or without FICZ. Gene expression was measured by RT-qPCR (*n* = 6 in 2 independent experiments). Two-way ANOVA. (**C**) Sca-1 expression in bone marrow cells. Representative flow cytometric results and MFI are shown. Mann-Whitney *U* test. (**D**) Sca-1 expression in immune cell types in blood. The median is shown (*n* = 4 in 2 independent experiments). Mann-Whitney *U* test. (**E**) Cytokine concentrations in plasma from Lysm*AhR^Δ^ mice (KO) and WT AhR^fl/fl^ littermates. The median is shown (*n* = 6 in 2 independent experiments). Mann-Whitney *U* test. (**F**–**I**) CD64*AhR^Δ^ mice (KO) and WT AhR^fl/fl^ littermates were used. (**F**) *AhR* expression was assessed ex vivo on monocytes from bone marrow, and *Ahrr* expression was assessed on monocytes cultured overnight with or without FICZ. Gene expression was measured by RT-qPCR. The median is shown (*n* = 4–7 in 2 independent experiments). Mann-Whitney *U* test for *Ahr*; 2-way ANOVA for *Ahrr*. (**G**) Peritoneal macrophages were cultured overnight with or without FICZ. Gene expression was measured by RT-qPCR. The median is shown (*n* = 8 in 2 independent experiments). Two-way ANOVA. (**H**) MFI of Sca-1 expression in bone marrow cells, with the median shown (*n* = 7 in 2 independent experiments). Mann-Whitney *U* test. (**I**) Plasma cytokine concentrations. The median is shown (*n* = 11 in 3 independent experiments). Mann-Whitney *U* test. (**J**–**L**) Cx3cr1*AhR^Δ^ mice (KO) and WT AhR^fl/fl^ littermates were analyzed 6 weeks after tamoxifen treatment. (**J** and **K**) MFI of Sca-1 expression in bone marrow cells. (**J**) Representative stainings. (**K**) MFI of Sca-1 expression in bone marrow cells, with the median shown (*n* = 10–11 in 2 independent experiments). Mann-Whitney *U* test. (**L**) Plasma concentrations of the indicated cytokines, with the median shown (*n* = 8–11 in 2 independent experiments). Mann-Whitney *U* test. For all panels, *P < 0.05, **P < 0.01, ***P < 0.001. Absence of asterisks indicates nonsignificance. Schematics in **A**, **F**, and **J** were created with BioRender.

To address whether this effect of AhR was specific to monocytes or shared with other mononuclear phagocytes, we crossed *AhR^fl/fl^* mice with *LysM*-cre mice. In LysM*AhR^Δ^ mice, we found that bone marrow monocytes were not deleted for *Ahr* and AhR activity was unaffected upon exposure to the agonist 6-formylindolo[3,2-b]carbazole (FICZ) as assessed by the expression of the canonical target gene *Ahrr* (Figure 3A). By contrast, macrophages were deficient for AhR activation, as shown by the decreased expression upon stimulation of AhR target genes *Ahrr* and *Cyp1b1* (Figure 3B). We detected neither upregulation of Sca-1 on immune cells in bone marrow (Figure 3C) or blood (Figure 3D) nor increased cytokine secretion in blood (Figure 3E), suggesting that AhR deficiency in macrophages does not increase homeostatic cytokine responses. To further confirm these observations, we generated another model for AhR deletion in macrophages by crossing *AhR^fl/fl^* mice with CD64-icre mice (40). In these mice, *AhR* expression and its activity were unaffected in monocytes (Figure 3F), while macrophages were deficient for AhR activation (Figure 3G). We found that Sca-1 expression on immune cells (Figure 3H) and blood cytokine levels (Figure 3I) were unchanged in CD64*AhR^Δ^ mice compared with WT littermates. Finally, we addressed the potential contribution of Cx3cr1-expressing macrophages to the phenotype observed in Cx3cr1*AhR^Δ^ mice. We induced *Ahr* deletion by tamoxifen treatment and then waited for 6 weeks before analysis. With this protocol, only long-lived macrophages remain deficient for *Ahr*, while short-lived monocytes regain normal *Ahr* expression (41). In this setting, Sca-1 expression on immune cells and blood cytokine levels were similar in deficient mice compared with WT littermates (Figure 3, J–L). Collectively, these results indicate that AhR is required to dampen tonic cytokine responses in monocytes but not in macrophages.

### AhR dampens STING-mediated tonic microbiota sensing.

**Figure 4:**
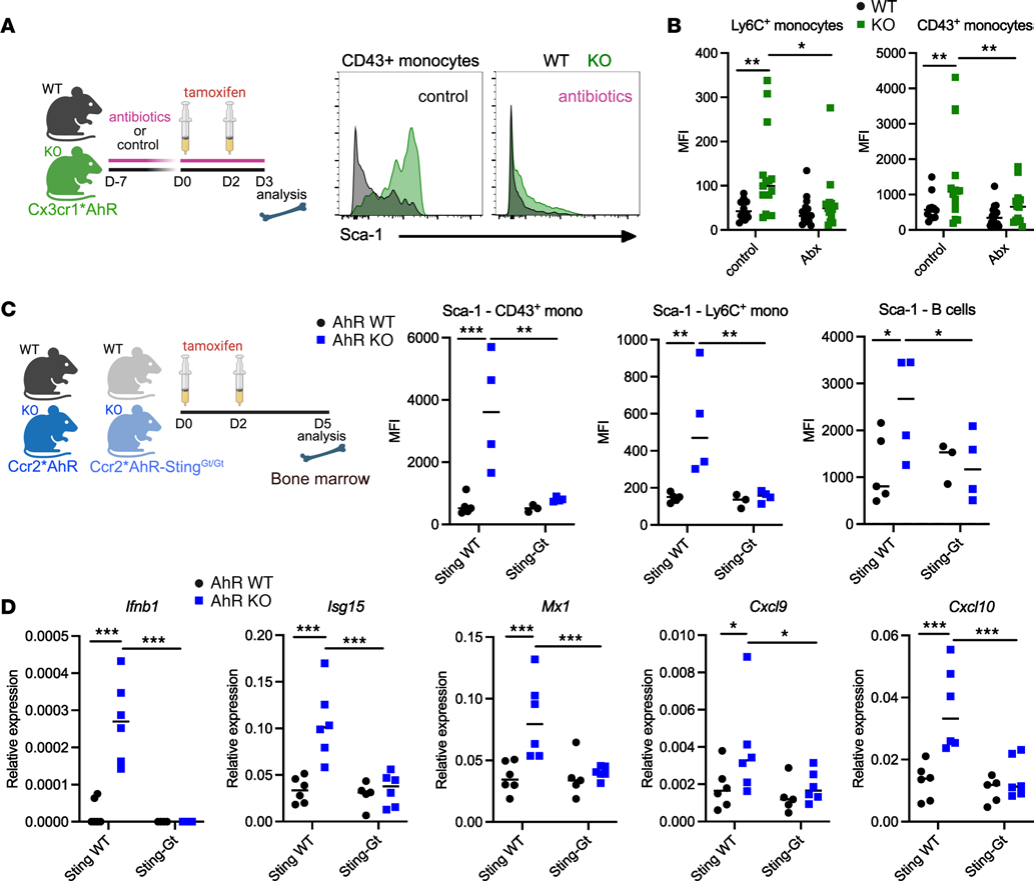
AhR dampens STING-mediated tonic microbiota sensing. (**A** and **B**) Cx3cr1*AhR^Δ^ mice (KO) and WT AhR^fl/fl^ littermates were treated orally with antibiotics for 1 week. Mice were analyzed after tamoxifen treatment. (**A**) Representative staining for Sca-1 expression in bone marrow CD43^+^ monocytes. (**B**) MFI, with the median shown (*n* = 13–15 in 5 independent experiments). Two-way ANOVA. (**C** and **D**) Ccr2*AhR^Δ^ (AhR-KO Sting^WT^), WT AhR^fl/fl^ (WT Sting^WT^), Ccr2*AhR^Δ^ Sting^Gt/Gt^ (AhR-KO Sting^Gt^), and WT AhR^fl/fl^ Sting^Gt/Gt^ (WT Sting^Gt^) mice were treated with tamoxifen. (**C**) MFI of Sca-1 expression in the indicated bone marrow cells. The median is shown (*n* = 3–5 in 2 independent experiments). Two-way ANOVA. (**D**) Bone marrow monocytes were purified. Expression of the indicated genes was assessed by RT-qPCR. The median is shown (*n* = 6 in 3 independent experiments). Two-way ANOVA. For all panels, *P < 0.05, **P < 0.01, and ***P < 0.001. An absence of asterisks indicates nonsignificance. The schematics in **A** and **C** were created with BioRender.

We then sought to assess whether tonic cytokine secretion was induced by sensing of microbiota-derived compounds. To this aim, we treated Cx3cr1*AhR^Δ^ mice and WT littermates with broad-spectrum antibiotics (metronidazole and neomycine) and assessed Sca-1 expression on monocytes as a readout for IFN-I responses (Figure 4, A and B). Of note, because ampicillin-sensitive microbiota produce AhR agonists (42), we did not use ampicillin to avoid depleting this physiological source of AhR stimulation. Antibiotics treatment significantly (Figure 4B) decreased Sca-1 expression in deficient mice, while it had no effect on WT littermates, suggesting that microbiota-derived compounds were the main source of tonic stimulation for inducing IFN-I production in monocytes. STING-dependent microbiota sensing was shown to be a major driver of tonic IFN responses in DCs (10). To address whether STING was involved in the tonic microbiota sensing that we observed in monocytes, we generated mice deficient for STING, in which *Ahr* could be deleted in monocytes upon tamoxifen treatment, by crossing Ccr2*AhR mice with Sting^Gt^ mice, which have a missense mutation of *Sting* functioning as a null allele (43). Increased expression of Sca-1 in immune cells (Figure 4C) and of *Ifnb1* and ISGs in monocytes (Figure 4D) was abolished in AhR-deficient monocytes when mice were also deficient for STING activation. Collectively, these results show that AhR activation in monocytes played an essential role in dampening STING-mediated tonic microbiota-induced responses.

We then sought to determine the main physiological source of AhR activation responsible for this phenomenon. To assess the role of diet-derived AhR ligands, we fed Cx3cr1*AhR^Δ^ mice and WT littermates a purified diet, which contains very low levels of indoles (indole-poor diet), therefore depleting the main source of diet-derived AhR agonists (44). We compared these groups with mice fed the same purified diet supplemented with indole-3-carbinole, a precursor of AhR agonists (I3C diet). We have previously shown that the I3C diet induces physiological AhR activation levels within the same range as those induced by a normal chow diet (44). In addition, to assess the contribution of the gut microbiota as a source of AhR agonists, we depleted mice fed an indole-poor or an I3C diet of ampicillin-sensitive species, among which *Lactobacilli* are major producers of indole-derivative AhR agonists (42, 45). Therefore we compared 4 groups of mice, depleted of either diet-derived or microbiota-derived AhR agonists, or both (Supplemental Figure 4, A and B). We found that reduced levels of diet-derived or of microbiota-derived AhR agonists did not increase ISG expression in monocytes or Sca-1 expression in immune cells in WT mice (Supplemental Figure 4, A and B), and only Cx3cr1*AhR^Δ^ mice showed increased tonic IFN responses, which remained similar across treatment conditions. These results suggest that AhR signaling can be activated in monocytes by other sources that are redundant with diet-derived and gut microbiota–derived AhR agonists, such as products of the kynurenine pathway (25).

### AhR activation limits tonic cytokine responses in human monocytes.

To address the relevance of our findings for humans, we analyzed blood monocytes from healthy donors. We reasoned that ex vivo exposure to culture medium would mimic physiological steady-state stimulation with AhR ligands. Indeed, tryptophan-derived AhR agonists are naturally present in culture medium (46), and added serum also contains circulating indole-derived AhR ligands (47). In addition, we have previously observed spontaneous TNF secretion by human monocytes starting from 3 hours after culturing (48), suggesting that culture medium contains a natural source of cytokine-inducing stimulus.

**Figure 5:**
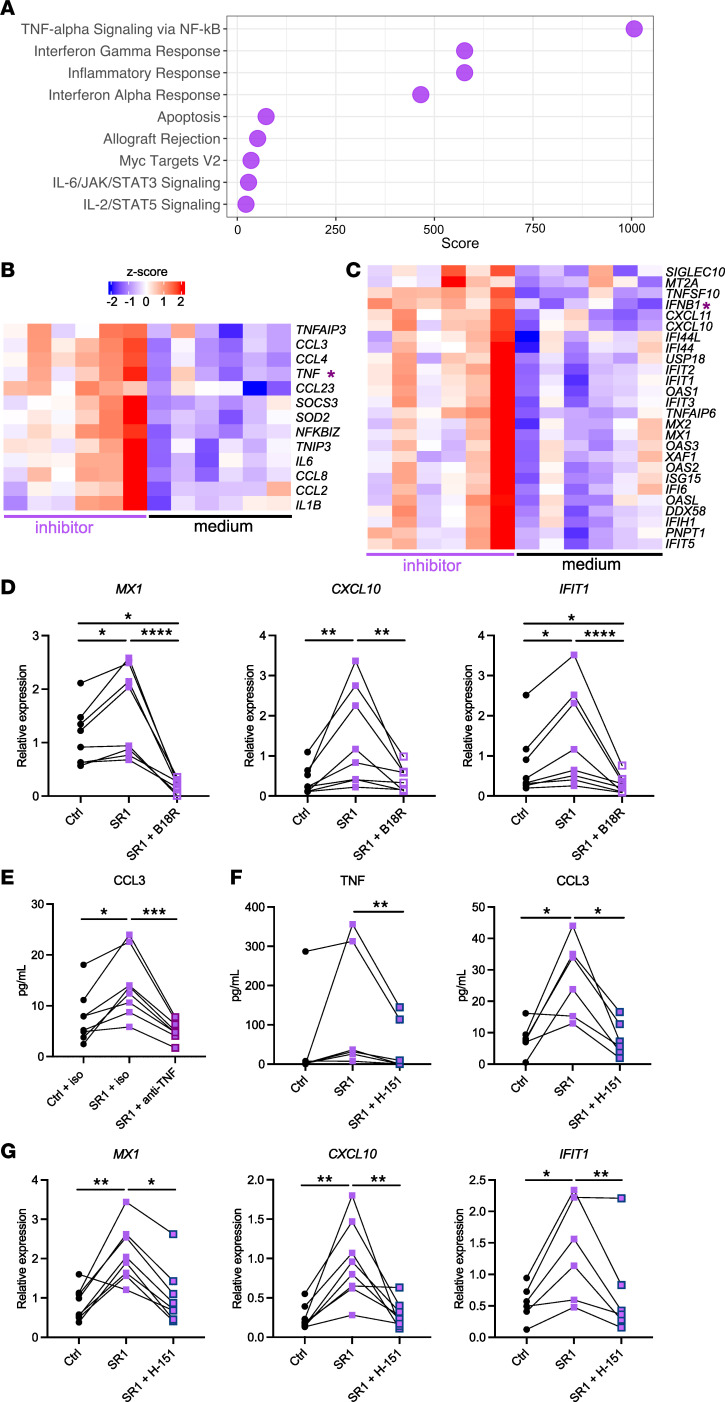
AhR activation limits tonic cytokine responses in human monocytes. Human blood monocytes were cultured for 6 hours in the presence or absence of the AhR inhibitor SR1. (**A**–**C**) Monocytes were subjected to RNA-Seq analysis (*n* = 6). (**A**) Enrichment analysis for GO biological pathways using genes differentially upregulated in SR1-treated monocytes. (**B** and **C**). Scaled expression of TNF-related (**B**) and IFN-stimulated (**C**) genes. *TNF* and *IFNB1* are highlighted by an asterisk. (**D**) Monocytes were cultured in the presence or absence of the IFNAR inhibitor B18R. Expression of the indicated genes was assessed by RT-qPCR. The median is shown (*n* = 8–9 in 4 independent experiments). (**E**) Monocytes were cultured in the presence of anti-TNF–blocking antibody or an isotype control (iso). The concentration of CCL3 in supernatant is shown (*n* = 6 in 3 independent experiments). (**F** and **G**) Monocytes were cultured in the presence or absence of the STING inhibitor H-151 (*n* = 8 in 4 independent experiments). (**F**) The concentration of the indicated cytokines in supernatant is shown. (**G**) Expression of the indicated genes was assessed by RT-qPCR. Ctrl, control condition with DMSO. *P < 0.05, **P < 0.01, ***P < 0.001, and ****P < 0.0001, by Friedman test.

To assess the role of AhR in response to homeostatic activation, we inhibited AhR signaling pharmacologically using stemregenin 1 (SR1) and performed RNA-Seq analysis after 6 hours. We focused on genes that were overexpressed with SR1 treatment compared with physiological activation (Supplemental Table 2). Pathway enrichment analysis showed an enrichment for TNF signaling and IFN responses (Figure 5A). Indeed, multiple TNF pathway genes (including *TNF*) (Figure 5B) and ISGs (including *IFNB1*) (Figure 5C) were overexpressed in the presence of SR1 compared with the medium condition, similar to our observations in mice deficient for AhR in monocytes. We hypothesized that ISG expression is a secondary effect due to an autocrine loop of IFN-β on monocytes. To directly test this, we assessed ISG expression by monocytes after exposure to a soluble IFN-I receptor (B18R) that acts as an inhibitor (49) (Figure 5D). SR1-augmented expression of *MX1*, *CXCL10*, and *IFIT1* was significantly diminished in the presence of B18R, showing that IFN-I signaling was required for ISG-increased expression upon AhR inhibition. We also tested whether expression of the TNF-related cytokine CCL3 was the result of an increased TNF autocrine loop, by using an anti-TNF–blocking antibody (Figure 5E). CCL3 secretion was increased in the presence of SR1, which was abolished with blockade of TNF. Collectively, these results indicate that physiological AhR activation represses spontaneous TNF and IFN-I production by human monocytes ex vivo.

Cytokine responses can be triggered by reactivation of endogenous retroviruses (ERVs) stimulating intracellular pathogen recognition pathways (50). We assessed the possibility that AhR inhibition would increase this phenomenon in monocytes, thereby stimulating cytokine secretion. To address this, we reanalyzed our transcriptomics data for transposable elements and specifically assessed ERV expression. While we detected the expression of various ERV elements in human monocytes cultured for 6 hours, there was no significant difference in ERV expression in SR1-treated monocytes compared with physiological condition (Supplemental Figure 5 and Supplemental Table 3). These results indicate that AhR inhibition does not increase ERV reactivation in human monocytes.

To test whether the STING pathway was involved in inducing the tonic cytokine production observed ex vivo in human monocytes, we inhibited STING activation using H-151, a potent selective STING antagonist (51). The increase in TNF and CCL3 secretion induced by SR1 was abolished in the presence of H-151 (Figure 5F). In addition, expression of *MX1*, *CXCL10*, and *IFIT1* was no longer upregulated by SR1 treatment when monocytes were exposed to H-151 (Figure 5G). These results suggest that in human monocytes, similar to the mouse, activation of AhR downmodulates STING-mediated tonic cytokine responses.

### Low physiological AhR activity in monocytes correlates with increased tonic cytokine responses in vivo in humans.

**Figure 6:**
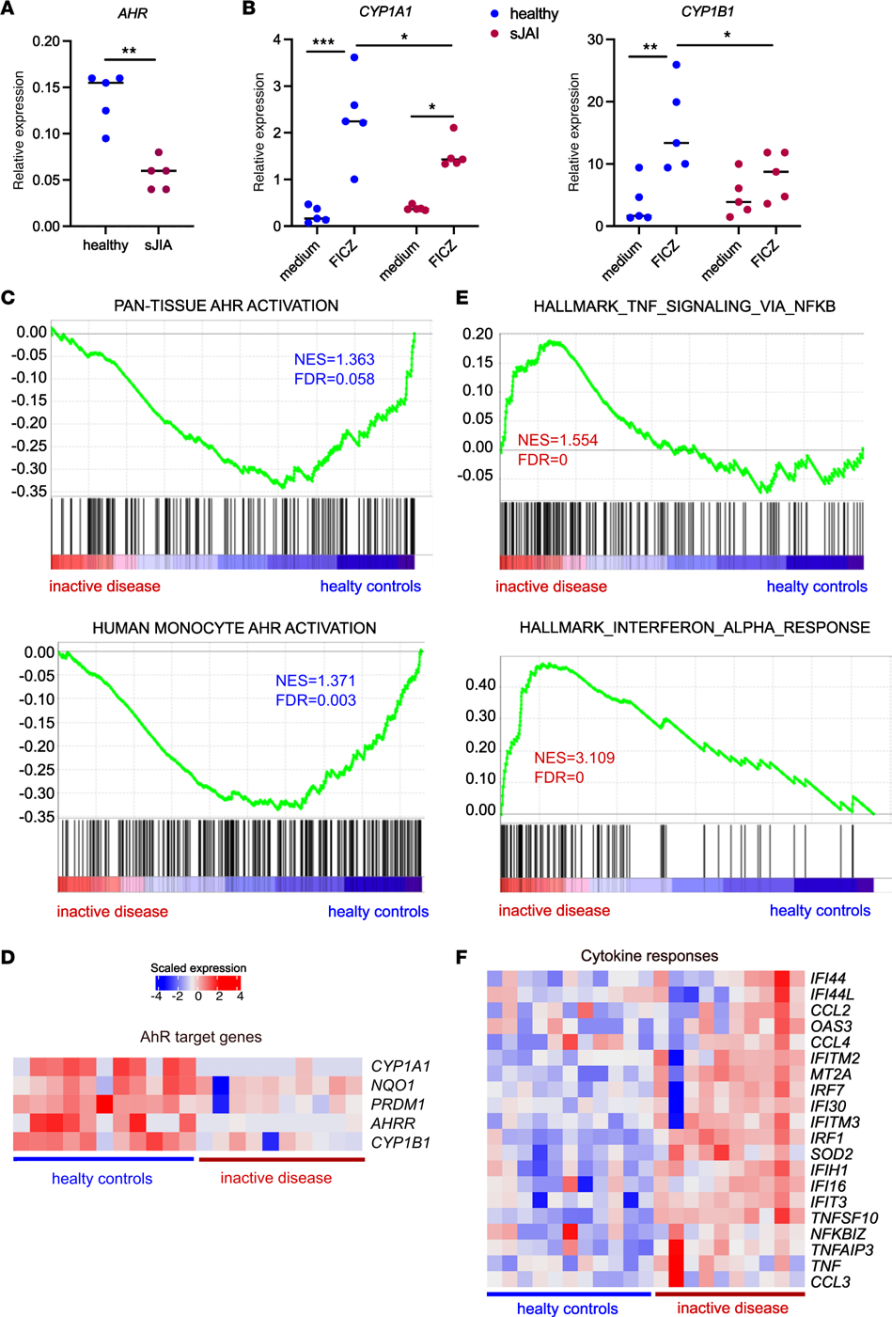
Low physiological AhR activation correlates with increased tonic cytokine responses in vivo in humans. (**A** and **B**) Monocytes from healthy controls and patients with sJIA with clinically inactive disease were isolated from blood. (**A**) *AhR* expression in monocytes was assessed by RT-qPCR (*n* = 5). Mann-Whitney *U* test. (**B**) Monocytes were cultured for 16 hours in the presence or absence of the AhR agonist FICZ. Expression of the indicated genes was assessed by RT-qPCR (*n* = 5). Two-way ANOVA. (**C**–**F**) Public transcriptomics data on blood monocytes (GSE147608) was reanalyzed, comparing data from healthy controls and patients with sJIA with clinically inactive disease. (**C** and **E**) GSEA was performed for healthy controls (blue) versus patients with inactive disease (red) for a pan-tissue AhR activation signature and our monocyte AhR activation signature (**C**), or Hallmark “TNF signaling via NF-κB” and Hallmark “interferon-alpha response” public signatures (**E**). The NES and FDR are indicated in the color of the enriched dataset. (**D** and **F**) Scaled expression of the indicated genes. For all panels, *P < 0.05, **P < 0.01, and ***P < 0.001.

To evaluate whether these findings apply to humans in an in vivo context, we analyzed monocytes from patients with systemic juvenile idiopathic arthritis (sJIA) who have clinically inactive disease. Indeed, we have previously shown that monocytes from patients with sJIA express low levels of AhR compared with healthy age-matched individuals (52) (Figure 6A). To assess AhR activity in these monocytes, we exposed them to the AhR agonist FICZ and measured the expression of canonical AhR target genes. Expression of *CYP1A1* and *CYP1B1* was significantly decreased in monocytes from patients with sJAI (Figure 6B), indicating lower AhR activity. We then reanalyzed transcriptomics data from purified blood monocytes from patients with sJIA (53). To assess AhR activity, we used a published “pan-tissue AhR activation” signature (54). We also designed our own signature for physiological AhR activation in human monocytes using the genes upregulated in the control medium condition versus those exposed to SR1. Gene set enrichment analysis (GSEA) showed significant enrichment for both gene signatures in monocytes from healthy individuals (Figure 6C). The list of genes and their contribution to the enrichment score are shown in Supplemental Table 4. In particular, AhR target genes were expressed at higher levels in monocytes from healthy controls (Figure 6D). Most of the human orthologs of genes enriched in steady-state WT mouse monocytes compared with AhR-deficient monocytes were also enriched in monocytes from healthy controls compared with monocytes from patients with sJIA (Supplemental Figure 6). These observations are consistent with decreased homeostatic AhR signaling in monocytes from patients with sJIA with inactive disease. By contrast, gene signatures for ISG and for TNF responses were significantly enriched in monocytes from sJIA patients with inactive disease (Figure 6E and Supplemental Table 4), including *TNF* and multiple ISGs (Figure 6F). These results show a correlation between low physiological AhR activity in monocytes and increased tonic cytokine responses in vivo in humans.

## Discussion

Here, we show a central role for monocytes in the homeostasis of tonic cytokine responses and demonstrate that AhR in monocytes is essential for dampening STING-induced microbiota sensing and avoiding excessive systemic cytokine production.

DCs, and in particular plasmacytoid DCs (pDCs), have been previously identified as the main sensors of microbiota-derived cues and producers of tonic IFN-I (6, 10, 11, 14, 15), but not other mononuclear phagocytes. Our results indicate that monocytes also sense microbiota-derived products capable of inducing IFN-β and TNF secretion. However, specifically in monocytes, tonic microbiota sensing is actively repressed by AhR signaling to restrain cytokine responses. This is consistent with observations in reporter mice that monocytes produce small amounts of IFN-β at steady state and that these amounts are significantly lower than those produced by pDCs (6). This is also in line with findings that microbiota-derived products are sensed by monocytes in the tumor context, inducing local production of IFN-β (55). Whether specific tumor-derived signals revert in monocytes, the dampening of microbiota sensing remains to be determined.

Previous work evidenced that AhR activation during viral infections suppresses IFN-I responses in macrophages (26, 27). We show here that AhR was also a roadblock in steady-state conditions to avoid excessive IFN-I responses. This phenomenon was essential in monocytes but dispensable in macrophages. It has been previously proposed that differences in chromatin accessibility render monocytes more sensitive than macrophages to microbial products (56), which may underlie the need for dampening tonic sensing specifically in monocytes. More work will be needed to decipher this cell-type specificity.

AhR is a ligand-activated transcription factor sensing a wide range of physiological ligands including tryptophan catabolites and indole derivatives generated from food products and microbiota metabolism (25). We found that decreased levels of diet-derived or gut microbiota–derived AhR ligands did not affect the control of tonic cytokine responses in monocytes. This indicates that this mechanism robustly resists perturbations and suggests a redundancy between different sources of AhR stimulation. However, AhR can also be activated by xenobiotics including pollutants and dioxins, which induce persistent AhR signaling by inhibiting its negative feedback loop (25). Whether such xenobiotics perturb tonic cytokine responses via their action on AhR deserves further attention.

We found that AhR in monocytes dampens STING-mediated tonic cytokine responses in both mice and humans. STING can activate IRF3 and NF-κB downstream signaling, resulting in IFN-I and TNF production, respectively (57), consistent with the dysregulation of both IFN-I– and TNF-driven cytokine responses we observed in vivo and in vitro. Interactions between STING and AhR have been previously reported in macrophages and tumor cells (31, 58, 59). AhR activation can interfere with the STING pathway indirectly by inducing the expression of TCDD-inducible poly (ADP-ribose) polymerase, which suppress the activity of TBK1, a downstream effector of STING (26, 58). AhR activation was also proposed to alter STING intra-cellular localization, thereby decreasing its activity (59). In another study, AhR was found to favor the ubiquitination and proteasome-dependent degradation of STING (31). Determining which of these mechanisms takes place in monocytes will require future investigation. In the mouse, STING-dependent microbiota sensing by DCs was shown to be mediated by microbiota-derived extracellular vesicles containing bacterial DNA (10). Here, in monocytes placed in culture, we also observed low constitutive STING-dependent cytokine production that was increased upon AhR inhibition. How the STING pathway becomes activated upon culture remains unclear. One possible trigger are microbiota-derived membrane vesicles that are naturally present in serum (60).

While tonic cytokine production is crucial for the development and conditioning of the immune system, the levels of circulating cytokines need to be tightly regulated to avoid systemic inflammation and autoimmunity. Our results suggest a division of labor between immune sentinels, with DCs acting as the main source of microbiota-driven tonic cytokines, while tonic monocyte activation is actively repressed to prevent excessive cytokine production.

## Methods

### Sex as a biological variable.

Both male and female mice were used. Sex as a biological variable was investigated in mouse experiments shown in Supplemental Figure 3. For humans, samples from both male and female donors were used. Sex was not considered as a biological variable for the human data.

### Mouse strains.

*Ccr2*-creER^T2^-mKate2 mice were described previously (32). *Lysm*-Cre (strain 004781), *Cx3cr1*-creER (strain 021160), *Ahr*-floxed (strain 006203), and Sting^Gt^ (strain 017537) mice were obtained from The Jackson Laboratory. *Fcgr1*-icre were obtained from Bernard Malissen (40). The Lysm*AhR strain was generated in-house by crossing *Lysm*-Cre and *Ahr*-flox mice. The Ccr2*AhR strain was generated in-house by crossing *Ccr2*-creER^T2^-mKate2 and *Ahr*-floxed mice. The Cx3cr1*AhR strain was generated in-house by crossing *Cx3cr1*-CreER and *Ahr*-floxed mice. The Cd64*AhR strain was generated in-house by crossing *Fcgr1*-icre and *Ahr*-floxed mice. The Ccr2*AhR-Sting^Gt^ strain was generated in-house by crossing Ccr2*AhR and Sting^Gt^ mice. All mice were on a C57BL/6 background. Mice were maintained under specific pathogen–free conditions at the animal facility of the Institut Curie in accordance with institutional guidelines. Mice were housed in a 12-hour light/12-hour dark environment, with ad libitum access to water and food. Unless otherwise indicated, the mice were fed a standard chow diet (4RF25 SV-PF 1609, Le Comptoir des Sciures). Cx3cr1*AhR female and male mice (AhR*^fl/fl^* Cre^+/–^ and Cre^–/–^ littermates), Lysm*AhR female and male mice (AhR*^fl/fl^* Cre^+/–^ and Cre^–/–^ littermates), Cd64*AhR female and male mice (AhR*^fl/fl^* Cre^+/–^ and Cre^–/–^ littermates), Ccr2*AhR female and male mice (AhR*^fl/fl^* Cre^+/+^ and Cre^–/–^ littermates), and Ccr2*AhR-Sting^Gt^ female and male mice (AhR*^fl/fl^*-Sting^Gt/Gt^ Cre^+/+^ and Cre^–/–^ littermates) were used and sacrificed at 8–12 weeks of age. For strains requiring tamoxifen exposure for gene deletion, all mice (Cre^+^ and Cre^–^) were treated in the same manner to avoid confounding effects from tamoxifen treatment. Unless otherwise indicated, Ccr2*AhR^Δ^ mice and WT littermates were treated twice with 5 mg tamoxifen (MilliporeSigma) resuspended in corn oil (MilliporeSigma) by oral gavage on day 0 and day 2 or day 3, and sacrificed on day 5. Unless otherwise indicated, Cx3cr1*AhR^Δ^ mice and WT littermates were treated with 5 mg tamoxifen (MilliporeSigma) resuspended in corn oil (MilliporeSigma) by oral gavage on 3 consecutive days and sacrificed on day 5 after the last treatment. In 1 experiment, Cx3cr1*AhR^Δ^ mice and WT littermates were sacrificed 6 weeks after the last day of treatment. To avoid confounding effects of circadian rhythms on monocyte biology, mice were always sacrificed for analysis at the same time of day (90 minutes after lights on). In 1 experiment, Cx3cr1*AhR^Δ^ mice and WT littermates were treated with 1 g/L veterinary neomycin and metronidazole diluted in drinking water with added sucrose (2 g/L). Antibiotics were administered continuously, starting 7 days before tamoxifen treatment. Sucrose (2 g/L) was added to the drinking water of the control groups. Mice were treated with 5 mg tamoxifen resuspended in corn oil by peritoneal injection on day 0 and day 2 and analyzed on day 3. In 1 experiment, Cx3cr1*AhR^Δ^ mice and WT littermates were fed a purified diet (indole-poor diet, AIN-93M, Safe Diets), or the same diet enriched at 200 ppm in indole-3-carbinol (I3C) (MilliporeSigma), termed the I3C diet. Mice were placed on the specific diet at 5 weeks of age and remained on the diet from there on. After a 2-week period of adaptation, the mice were also treated with 1 g/L veterinary ampicillin (Ampisol, Dopharma France) diluted in drinking water with added sucrose (2 g/L), or sucrose alone (2 g/L) added to the drinking water for the control groups. After 1 week of ampicillin treatment, the mice were treated with tamoxifen as described above.

### Human samples.

Buffy coats from healthy donors (both male and female donors) were obtained from Etablissement Français du Sang (Paris, France). Children and adolescents with sJIA were enrolled at the Rheumatology Clinic at Texas Scottish Rite Hospital for Children. The characteristics of the 5 patients were as follows: 3 were male participants, aged 15, 17, and 18 years, and 2 were female participants, aged 15 and 17 years. Clinical laboratory measurements obtained in the clinic at sampling included a CBC and measurement of the erythrocyte sedimentation rate and liver enzyme levels. Patients with sJIA were considered “untreated,” as they did not receive any therapy other than nonsteroidal antiinflammatory drugs. The patients were considered to be inactive according to the physician’s clinical examination and a null MD Global score (a physician’s assessment of overall disease activity with a range of 0–10). Inactive, untreated patients fulfilled the remission-off-medication criteria for juvenile idiopathic arthritis including sJIA (61). Healthy control participants were enrolled either at Baylor University Medical Center (Dallas, Texas, USA) or the Etablissement Français du sang (Paris, France) and did not report any acute or chronic illness, were not receiving immunomodulatory therapies, and had not received a vaccine at least 1 month before sampling was done. The 2 female participants were 18 and 20 years old (Baylor Institute, Dallas, Texas, USA), and the 3 male participants were 20, 21 and 24 years old (Paris, France).

### Flow cytometry.

Cells were stained with the indicated antibody cocktails supplemented with Fc block (BD Biosciences) in FACS buffer (PBS containing 0.5% BSA and 2 mM EDTA) for 30–45 minutes on ice. For dead cell exclusion, cells were either stained with LIVE/DEAD fixable blue dead dye (Thermo Fisher Scientific, dilution 1:1,000) prior to antibody staining or were resuspended after staining in FACS buffer containing DAPI (Fischer Scientific, 100 ng/mL). Cells were acquired on a ZE5 instrument (Bio-Rad). Supervised analysis was performed using FlowJo software, version 10.

### Bone marrow and blood cell analysis.

Bone marrow cells were flushed out from legs and filtered using 40 mm cell strainers. Ten percent of the bone marrow cells or 50 mL blood was analyzed. Bone marrow and blood cell suspensions were treated with RBC lysis buffer (MilliporeSigma) for 5 minutes at room temperature.

Cells were stained with anti–TCRb BUV737 (BD Bioscience, clone H57-597); anti–Sca1 BV421 (BioLegend, clone D7); anti–CD19 BV480 (BD Bioscience clone 1D3); anti–Ly6G BV605 (BioLegend, clone 1A8); anti–CCR2 BV711 (BD Bioscience, clone 475301); anti–CD43 BB700 (BD Bioscience, clone S7); anti–CD11b PeDazzle594 (BD Bioscience, clone M1/70); anti–Ly6C PeCy7 (BioLegend, clone HK1.4); and anti–CD115 APC (BD Bioscience, clone AFS98).

### Splenic cell analysis.

Spleens were cut into small pieces with scalpels and digested for 30 minutes at 37°C in a digestion mix of RPMI containing 0.4 mg/mL DNAse I (MilliporeSigma) and 0.5 mg/mL collagenase D (Roche). Splenic cell suspensions were then filtered using 40 mm cell strainers and treated with RBC lysis buffer for 5 minutes at room temperature.

Cells were stained with anti–TCRb BUV737 (BD Bioscience, clone H57-597); anti–CD19 BV480 (BD Bioscience, clone 1D3); anti–Sca1 BV421 (BioLegend, clone D7); anti–Ly6G BV605 (BioLegend, clone 1A8); anti–CD11c BV786 (BD Biosciences, clone HL3); anti–Ly6C PeCy7 (BioLegend, clone HK1.4); anti–CD11b PerCPCy5.5 (BD Bioscience, clone M1/70); and anti–MHC II APC Cy7 (BioLegend, clone M5/114.15.2).

### Mouse cell purification and culturing.

Splenic cells were recovered by dissociation through 40 mm cell strainers. NK cells were purified with the EasySep Mouse NK Cell Isolation Kit (STEMCELL Technologies) according to the manufacturer’s recommendations. Cells were 80%–90% pure as verified by flow cytometry.

Peripheral lymph node cells were recovered by dissociation through 40 mm cell strainers. Total T cells were purified from with the EasySep Mouse T Cell Isolation Kit (STEMCELL Technologies) according to the manufacturer’s recommendations. Cells were 80%–90% pure as verified by flow cytometry.

Bone marrow cells were flushed out from legs. Monocytes were purified with the EasySep Mouse Monocyte Isolation Kit (STEMCELL Technologies) according to the manufacturer’s recommendations. Cells were 90% pure as verified by flow cytometry, with approximately 5% neutrophil contamination and less than 1% CD43^+^ monocytes (62).

Peritoneal lavage was recovered by i.p. injection of 5 mL PBS. Peritoneal macrophages were purified with the EasySep Mouse Monocyte Isolation Kit (STEMCELL Technologies) according to the manufacturer’s recommendations. Cells were 80%–90% pure as verified by flow cytometry.

For analysis of AhR activity, cells were cultured for 16 hours with RPMI GlutaMAX (Gibco, Thermo Fisher Scientific) supplemented with antibiotics (penicillin and streptomycin) and 10% FCS (Serana), alone or in the presence of 60 nM 6-formylindolo[3,2-b]carbazole (FICZ) (Enzo Life Sciences).

### Cytokine measurement.

For cytokine measurement in mice, blood was collected and left at room temperature for 3 hours to coagulate. After centrifugation (450*g*, 10 minutes), plasma was collected for analysis and kept at –20°C. TNF and IL-6 concentrations were measured using Enhanced Sensitivity CBA (BD Biosciences), with a limit of detection of 274 fg/mL. CCL2 and CXCL9 concentrations were measured using mouse CBA (BD Biosciences), with a limit of detection of 10 pg/mL.

For human cytokine detection, monocyte culture supernatants were collected and kept at –20°C. TNF and CCL3 concentrations were measured using human CBA (BD Biosciences), with a limit of detection of 10 pg/mL.

### Human monocyte purification and culturing.

PBMCs were obtained by centrifugation on a Ficoll gradient (Lymphoprep, STEMCELL Technologies). Blood monocytes were then positively isolated using CD14^+^ microbeads (Miltenyi Biotec) according to the manufacturer’s recommendations. Cells were 92%–95% pure as verified by flow cytometry. For RNA-Seq analysis, human monocytes were cultured for 6 hours with RPMI-GlutaMAX (Gibco, Thermo Fisher Scientific) supplemented with antibiotics (penicillin and streptomycin) and 10% FCS, alone or in the presence of 8 mM SR1 (Cayman Chemicals). In some experiments, monocytes were cultured in the presence of 1 mg/mL recombinant B18R (STEMCELL Technologies), or after 90 minutes before exposure to 4 mg/mL H-151 (InvivoGen), or in the presence of 60 nM FICZ. DMSO was included in the control medium condition at a similar concentration.

### RNA-Seq library preparation.

Cells were lysed in RLT buffer (QIAGEN). Total RNA was extracted using the RNAeasy Mini kit (QIAGEN) including on-column DNase digestion according to the manufacturer’s protocol. The integrity of the RNA was confirmed in BioAnalyzer using the RNA 6000 Nano kit (Agilent Technologies) (8.8 <RIN <10). Libraries were prepared according to Illumina’s instructions accompanying the TruSeq Stranded mRNA Library Prep Kit (Illumina). RNA (500 ng) was used for each sample. Library length profiles were controlled with the LabChip GXTouchHT system (PerkinElmer). Sequencing was performed using NovaSeq 6000 (Illumina) (100 nt length reads, paired-end).

### RNA-Seq data analysis.

Genome assembly was based on the Genome Reference Consortium (mm10 for mouse and hg38 for human). The quality of RNA-Seq data was assessed using *FastQC*. Reads were aligned to the transcriptome using *STAR* (63). Differential gene expression analysis was performed using *DESeq2* (version 1.22.2) (64). Genes with low count numbers (<10) were filtered out. For mouse monocytes, genes with differential expression between WT and KO conditions were calculated using the design group. For human monocytes, genes with differential expression between SR1 and medium treatments were calculated using the design donor + group. Differentially expressed genes were identified on the basis of an adjusted *P* value of less than 0.01 and a log_2_ fold change of greater than 0.5. Complete gene lists are included in Supplemental Tables 1 and 2. Heatmaps of log_2_-scaled expression were generated with ComplexHeatmap. Pathway enrichment was analyzed in the Molecular Signatures Database (MSigDB) (version 7.5.1) (65) using EnrichR (66).

### Analysis of transposable elements.

Transposable element (TE) expression was quantified using featureCounts from the Subread Suite package (version 2.0.6) with the following parameters: -p -M --primary –ignoreDup for individual TE counts. The annotation file used for TE quantification was from TEtranscripts (https://labshare.cshl.edu/shares/mhammelllab/www-data/TEtranscripts/TE_GTF/hg38_rmsk_TE.gtf.gz). The TE raw count matrix was imported to R (version 4.3.2). Transcripts per million (TPM) values were calculated using the tpm() function from *DRnaSeq* (version 1.0.0). Individual TEs were kept if detected in at least 2 samples from 1 group (medium or SR1) and if their mean TPM across all samples was above to 1. Only individual TEs passing these thresholds were kept in the raw count matrix. We used the removeBatchEffect() function from limma (version 3.56.2) to account for differences associated with the sex of the healthy donors. After correction, counts were normalized with the vst() function, and differential expression analysis was performed using *DESeq2* (version 1.40.2). Multiple testing correction was performed using the Benjamini-Hochberg method, and TEs with an adjusted *P* value below 0.05 were considered to be differentially expressed between conditions. TEs of the long terminal repeat (LTR) class were selected by applying the dplyr:filter() function on the class annotation. Hierarchical clustering based on Euclidean distances for samples and TEs was performed and is represented as a scaled heatmap using ComplexHeatmap (version 2.16.0). Complete lists of differentially expressed LTR elements are provided in Supplemental Table 3.

### Quantitative PCR.

Cells were lysed in RLT buffer (QIAGEN). RNA extraction was carried out using the RNAeasy Micro kit (QIAGEN) according to the manufacturer’s instructions. Total RNA was retrotranscribed using Superscript II Polymerase (Invitrogen, Thermo Fisher Scientific), in combination with random hexamers, oligo (dT), and deoxynucleotide triphosphates (dNTPs) (Promega). Transcripts were quantified by real-time PCR on a 480 LightCycler instrument (Roche). Reactions were carried out in 10 mL using Takyon Mastermix (Eurogentec) with the following TaqMan assay primers (Merck) for mouse samples: *Ahr* (Mm00478932_m1), *Gapdh* (Mm99999915_g1), *Polr2a* (Mm00839502_m1), *Isg15* (Mm01705338_s1), *Mx1* (Mm00487796_m1), *Cxcl9* (Mm00434946_m1), *Cxcl10* (Mm00445235_m1), *Ifnb1* (Mm00439552_s1), *Ahrr* (Mm00477443_m1), *Cyp1b1* (Mm00487229_m1). The following TaqMan assay primers (Merck) were used for human samples: *B2M* (Hs99999907_m1), *RPL34* (Hs00241560_m1), *HPRT1* (Hs02800695_m1), *CYP1A1* (Hs01054796_g1), *CYP1B1* (Hs00164383_m1), *MX1* (Hs00895608_m1), *IFIT1* (Hs03027069_s1), *CXCL10* (Hs00895608_m1). The second derivative method was used to determine each Cp (a Cp corresponds to the first peak of a second derivative curve), and the expression of genes of interest relative to the housekeeping genes (*Gapdh* and *Polr2a* for mouse, *B2M*, *HPRT1*, and *RPL34* for human) was quantified.

### GSEA.

Transcriptomics data were extracted from a the Gene Expression Omnibus (GEO) database (GSE147608) for blood monocytes from age-matched healthy controls and patients with systemic juvenile idiopathic arthritis with clinically inactive disease. Inactive disease was defined in the original study by the Wallace criteria (67). GSEA (68) was performed using GSEA software (version 4.0.3) with the default parameters, except for the number of permutations that we fixed at *n* = 1,000. Results were considered significant when the normalized enrichment score (NES) was greater than 1 and the FDR less than 0.25. Gene signatures for Hallmark “TNF signaling via NF-κB” and “interferon-a response” were downloaded from the MSigDB (version 7.5.1).

### Statistics.

Statistical tests were performed using GraphPad Prism 10 (GraphPad Software). Statistical details for each experiment can be found in the corresponding Figure legends. A P value of less than 0.05 was considered significant. Two-way ANOVA was performed with Šídák’s multiple-comparison test. The *n* value corresponds to the number of biological replicates analyzed (individual mice or human donors).

### Study approval.

All animal procedures were in accordance with the guidelines and regulations of the French Veterinary Department and have been approved by the local ethics committee (CEEA118 Comité Recherche et Ethique Animale de l’institut Curie, Paris, France) (authorization APAFIS no. 25217-2020042522586261 v1). According to French Public Health Law (art L 1121-1-1, art L 1121-1-2), written consent and IRB approval are not required for human noninterventional studies.

The study protocol for patients with sJIA was approved by the IRBs at Baylor University Medical Center (011-200, 007-221, 012-200), the University of Texas Southwestern Medical Center (092010–167), and the Texas Scottish Rite Hospital (09-11-060). Written informed consent was obtained from adults and the parents or guardians of those younger than 18 years of age. Buffy coats from healthy donors (both male and female donors) were obtained from the Etablissement Français du Sang (Paris, France) in accordance with INSERM ethics guidelines.

### Data availability.

Sequencing data have been deposited in GEO database (GEO GSE209803 for mouse and GSE214091 for human data). Experimental data are available is the Supporting Data Values file.

## Author contributions

Investigation: AR, A Cros, ADJ, A Coillard, MRL, DTO, and ES performed experiments. ES conceptualized the study. AR, A Cros, ADJ, A Coillard, EP, and ES conducted formal analysis. AR, CG, MB, and ES designed the study methodology. AMC, RB, VP, and BB provided essential tools. ES supervised the work. Writing – original draft: AR and ES wrote the original draft of the manuscript. All authors reviewed and edited the manuscript. Co–first author position was assigned by alphabetical order.

## Funding support

This work is the result of NIH funding, in whole or in part, and is subject to the NIH Public Access Policy. Through acceptance of this federal funding, the NIH has been given a right to make the work publicly available in PubMed Central.

- INSERM, Institut Curie, Cancéropôle Ile-de-France, Institut National du Cancer (2018-1-PLBIO-01-ICR1).
- NIH (P50 AR054083-01, U19 AIO82715).
- Agence Nationale de la Recherche (ANR-10-LABX-0043, ANR-10-IDEX-0001-02 PSL, ANR-17-CE15-0011-01, ANR-23-CE15-0033-03).
- Paris Ile-de-France Region-DIM ITAC (funding to MB and EP).

## Supplementary Material

Supplemental data

Supplemental table 1

Supplemental table 2

Supplemental table 3

Supplemental table 4

Supporting data values

## Version 1 10/02/2025

In-Press Preview

## Version 2 12/01/2025

Electronic publication
