# Supplementary material for: Aryl hydrocarbon receptor restrains tonic cytokine responses by inhibiting microbiota sensing in monocytes
Source: J Clin Invest. 2025 Oct 2;135(23):e189937. doi: 10.1172/JCI189937 (PMC12646677; doi:10.1172/JCI189937)
Supplement: Supplemental data [file jci-135-189937-s023.pdf]

Supplementary material

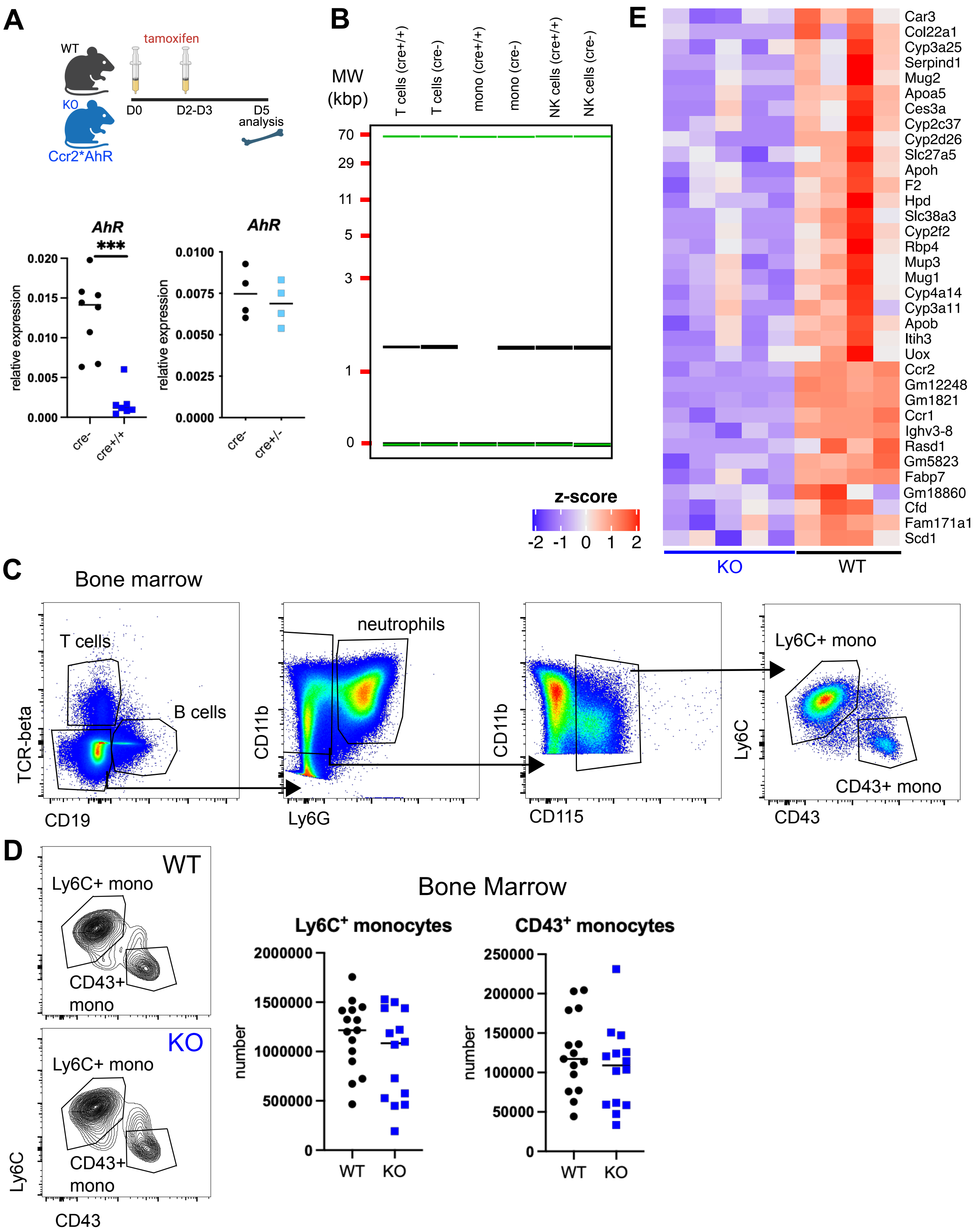

Supplementary figure 1. Characterization of Ccr2*AhR mice. Mice were treated with tamoxifen. **(A)** Monocytes from AhR$^{fl/fl}$ (cre-), Ccr2-cre$^{+/-}$ AhR$^{fl/fl}$ (cre+/-) or Ccr2-cre$^{+/+}$ AhR$^{fl/fl}$ (cre+/+) littermates were analyzed. *AhR* expression was measured by

RT-qPCR. Median is shown (n=7-8 for cre- vs cre+/+ in 2 independent experiments and n=4 for cre- vs cre+/- in 2 independent experiments). Mann-Whitney test, *** $p<0.001$. **(B-E)** Ccr2*AhR$^{\Delta}$ (KO, cre+/+) mice and AhR$^{fl/fl}$ littermates (WT, cre-) were used. **(B)** Monocytes were purified from the bone marrow, T cells from peripheral lymph nodes and NK cells from the spleen. Genomic PCR for AhR. Molecular ladder (MW) is shown. **(C)** Gating strategy for analysis of bone marrow cells. Cells were gated on live singlets. Representative results for WT AhR$^{fl/fl}$ mice are shown. mono=monocytes. **(D)** Representative flow cytometry results are shown. Numbers of indicated cell types in steady-state bone marrow of Ccr2*AhR$^{\Delta}$ mice or WT AhR$^{fl/fl}$ littermates. Numbers in bone marrow from one leg. Median is shown (n=14-15 in 5 independent experiments). Mann-Whitney test. Absence of stars indicates ‘not significant’. **(E)** Monocytes were purified from the bone marrow and were subjected to RNA-seq analysis (n=4-5 biological replicates). Scaled expression of differentially up-regulated genes in WT monocytes compared to AhR-deficient monocytes.

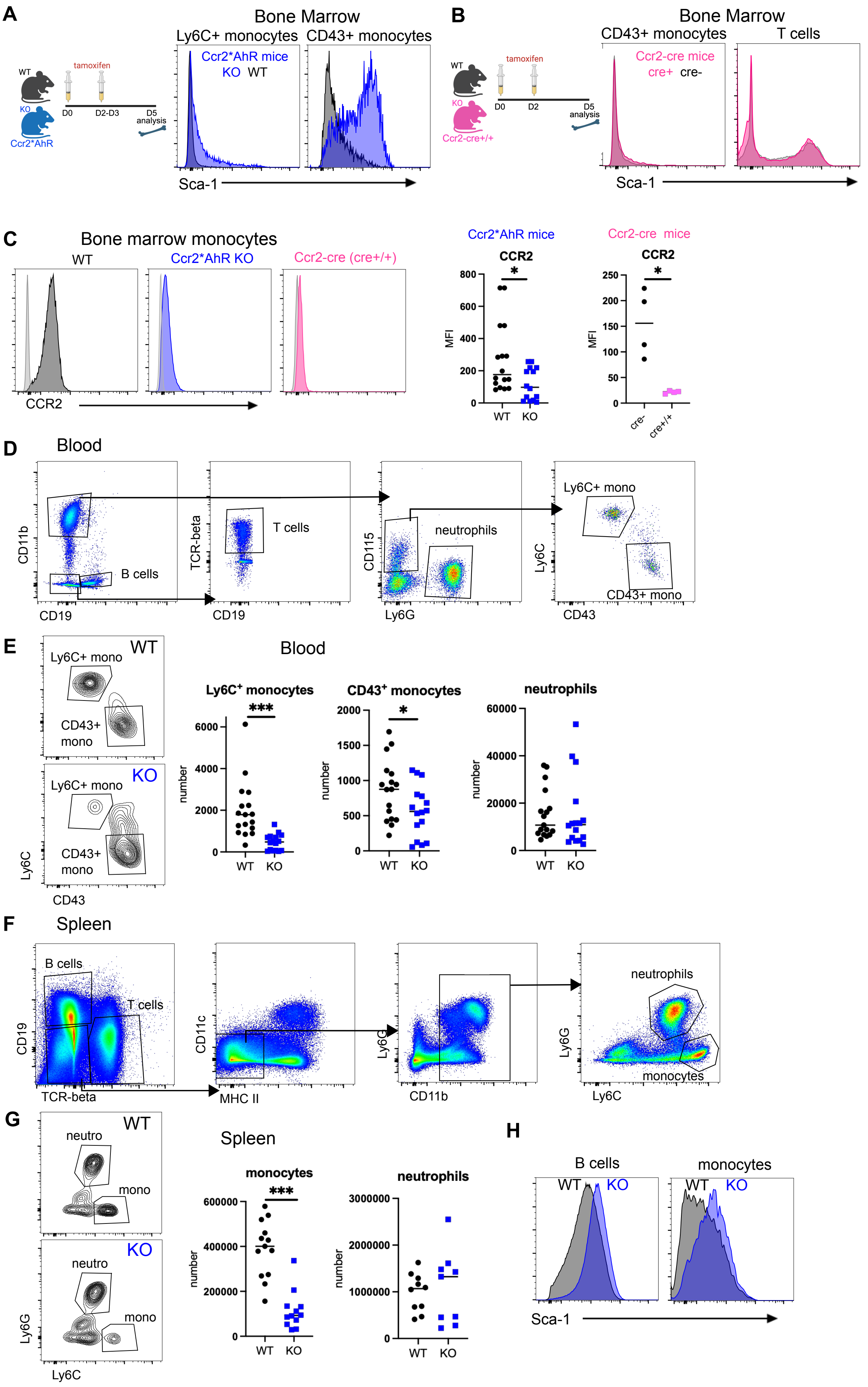
A
Bone Marrow
Ly6C+ monocytes
CD43+ monocytes
WT
KO
Ccr2*AhR
tamoxifen
D0
D2-D3
D5 analysis
Ccr2*AhR mice
KO WT
Sca-1
B
Bone Marrow
CD43+ monocytes
T cells
WT
KO
Ccr2-cre+/+
tamoxifen
D0
D2
D5 analysis
Ccr2-cre mice
cre+ cre-
Sca-1
C
Bone marrow monocytes
WT
Ccr2*AhR KO
Ccr2-cre (cre+/+)
CCR2
Ccr2*AhR mice
CCR2
MFI
WT
KO
Ccr2-cre mice
CCR2
MFI
cre-
cre+/+
D
Blood
CD11b
CD19
B cells
TCR-beta
CD19
T cells
CD115
Ly6G
neutrophils
Ly6C
CD43
Ly6C+ mono
CD43+ mono
E
Blood
WT
Ly6C+ mono
CD43+ mono
KO
Ly6C+ mono
CD43+ mono
Ly6C
CD43
Ly6C+ monocytes
CD43+ monocytes
neutrophils
number
WT
KO
F
Spleen
B cells
T cells
CD19
TCR-beta
CD11c
MHC II
Ly6G
CD11b
neutrophils
monocytes
Ly6G
Ly6C
G
WT
neutro
mono
KO
neutro
mono
Ly6G
Ly6C
Spleen
monocytes
neutrophils
number
WT
KO
H
B cells
monocytes
WT KO
WT KO
Sca-1

Supplementary figure 2. Ccr2*AhR mice with hypomorphic expression of Ccr2 display reduced numbers of circulating monocytes. (A-H) Ccr2*AhR (KO, cre+/+) mice and $AhR^{fl/fl}$ littermates (WT) were treated with tamoxifen. **(A)** Sca-1 expression on bone marrow monocytes. Representative flow cytometry results. **(B-C)** Ccr2-cre^{RT2} cre+/+ mice and cre- littermates were treated with tamoxifen. **(B)** Sca-1 expression on bone marrow CD43+ monocytes and T cells. Representative flow cytometry results (n=4 in 2 independent experiments). **(C)** CCR2 expression on bone marrow monocytes from Ccr2*AhR^{Δ} KO mice or WT $AhR^{fl/fl}$ littermates or Ccr2-cre^{RT2} cre+/+ mice. Representative flow cytometry results. Light grey histograms represent fluorescence-minus-one controls. Mean Fluorescence intensity is represented. Median is shown (n=16 in 5 independent experiments for Ccr2*AhR and n=4 in 2 independent experiments for Ccr2-cre^{RT2} mice). Mann-Whitney test. **(D)** Gating strategy for analysis of blood cells. Cells were gated on live singlets. Representative results for WT $AhR^{fl/fl}$ mice are shown. **(E)** Representative flow cytometry results are shown. Numbers of indicated cell types in 50 μL of blood. Median is shown (n=16-17 in 5 independent experiments). Mann-Whitney test. **(F)** Gating strategy for analysis of spleen cells. Cells were gated on live singlets. Representative results for WT $AhR^{fl/fl}$ mice are shown. **(G)** Representative flow cytometry results are shown. Numbers of indicated cell types in spleen. Median is shown (n=12-13 in 4 independent experiments). Mann-Whitney test. **(H)** Sca-1 expression on indicated spleen cells. Representative flow cytometry results. For all panels,* $p<0.05$, ** $p<0.01$, *** $p<0.001$. Absence of stars indicates 'not significant'.

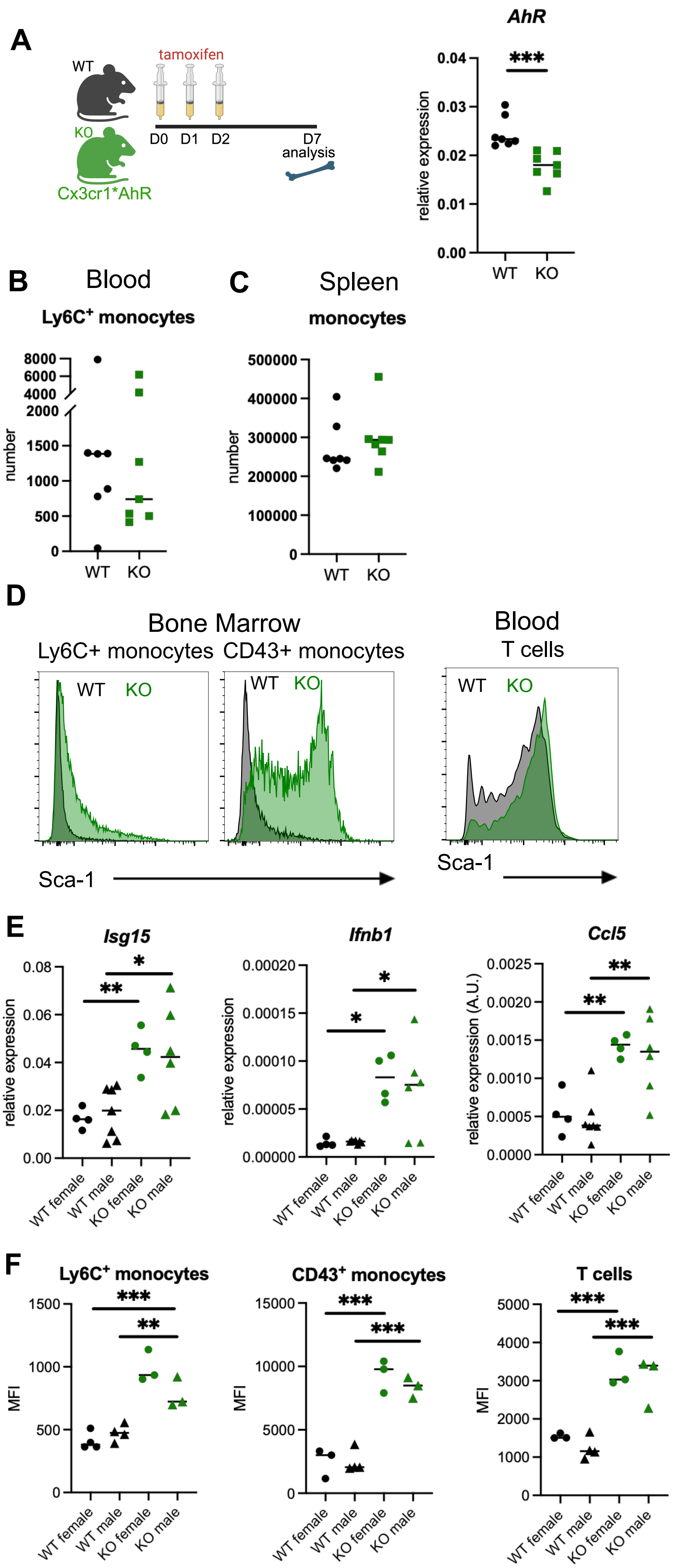

A
WT
KO
tamoxifen
D0 D1 D2
D7
analysis
Cx3cr1*AhR
AhR
***
relative expression
WT KO
B
Blood
Ly6C+ monocytes
number
WT KO
C
Spleen
monocytes
number
WT KO
D
Bone Marrow
Ly6C+ monocytes CD43+ monocytes
WT KO
Blood
T cells
Sca-1
E
Isg15
Ifnb1
Ccl5
relative expression
relative expression (A.U.)
WT female
WT male
KO female
KO male
F
Ly6C+ monocytes
CD43+ monocytes
T cells
MFI

Supplementary figure 3. Deficient AhR expression in monocytes causes dysregulation of tonic cytokine responses in vivo in both male and female. Cx3cr1*AhR$^{\Delta}$ mice (KO) and WT AhR$^{fl/fl}$ littermates were treated with tamoxifen. **(A and E)** Monocytes were purified from the bone marrow. *AhR* expression was measured by RT-qPCR. Median is shown (n=7-8 in 2 independent experiments). Mann-Whitney test. **(B-C)** Number of monocytes in 50 μL of blood (B) and in spleen (C). **(D)** Sca-1 expression in immune cell types of indicated tissues from Cx3cr1*AhR$^{\Delta}$ mice or WT AhR$^{fl/fl}$ littermates. Representative flow cytometry results. **(E)** Expression of indicated genes was assessed by RT-qPCR in monocytes from male or female mice. Median is shown (n=4-7 in 2 independent experiments). Brown-Forsythe and Welch ANOVA test. **(F)** Sca-1 expression in immune cell types in bone marrow from male and female mice. Two-way ANOVA.

For all panels,*** p<0.001. Absence of stars indicates 'not significant'.

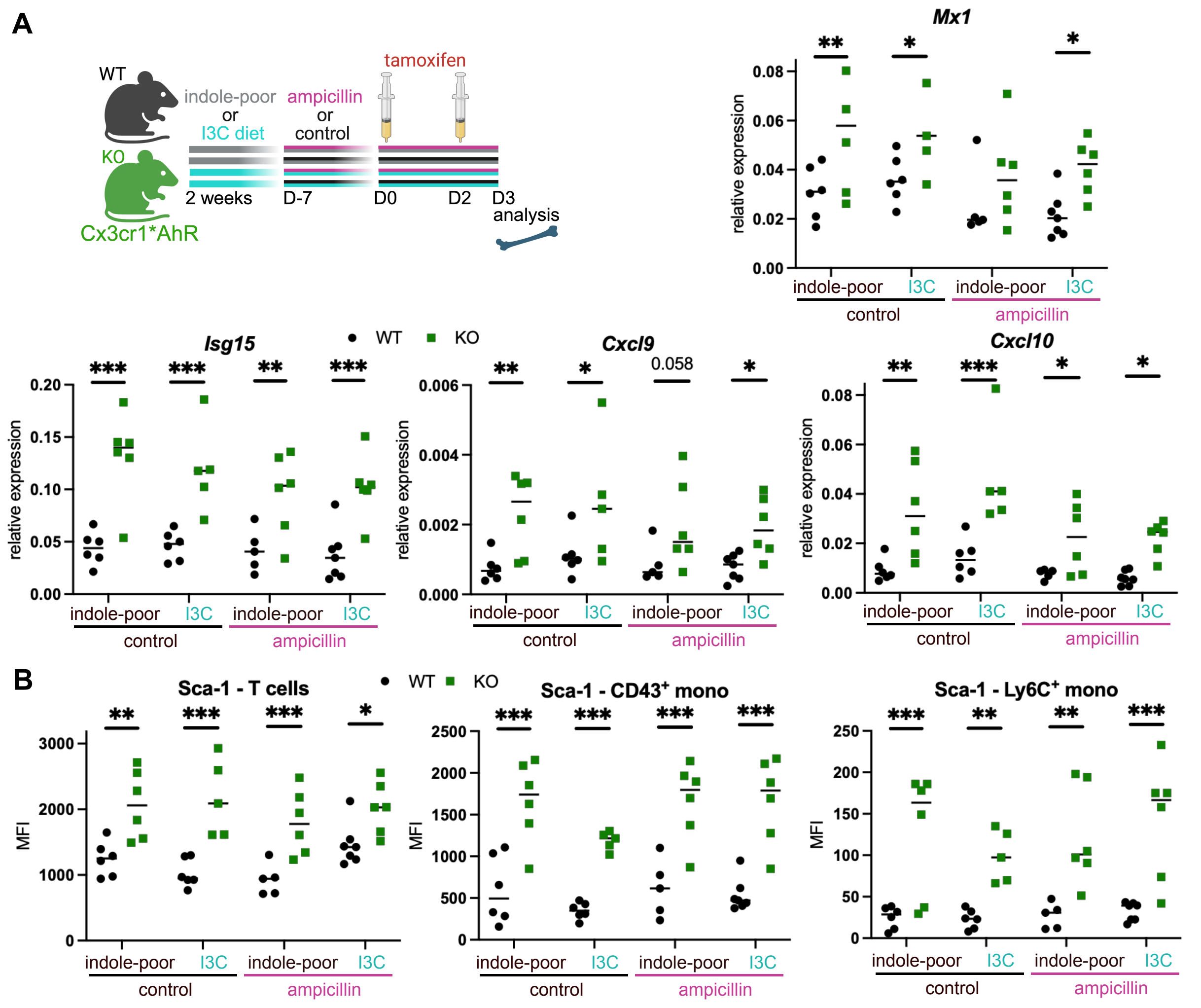

Supplementary figure 4. Reduced levels of diet-derived or gut microbiota-derived AhR agonists do not impact tonic interferon responses in monocytes. Cx3cr1*AhR$^{\Delta}$ mice (KO) and WT AhR$^{fl/fl}$ littermates were fed on indole-poor or I3C diets for 2 weeks. Mice were then treated or not with ampicillin for one week. Mice were analyzed after tamoxifen treatment. **(A)** Expression of indicated genes was assessed by RT-qPCR in bone marrow monocytes. Median is shown (n=5-7 in 2 independent experiments). Two-way ANOVA. **(B)** Sca-1 expression in indicated immune cell types from bone marrow (Mean Fluorescence Intensity, MFI). Median is shown (n=5-7 in 2 independent experiments). Two-way ANOVA. For all panels,* $p<0.05$, ** $p<0.01$, *** $p<0.001$. Absence of stars indicates ‘not significant’.

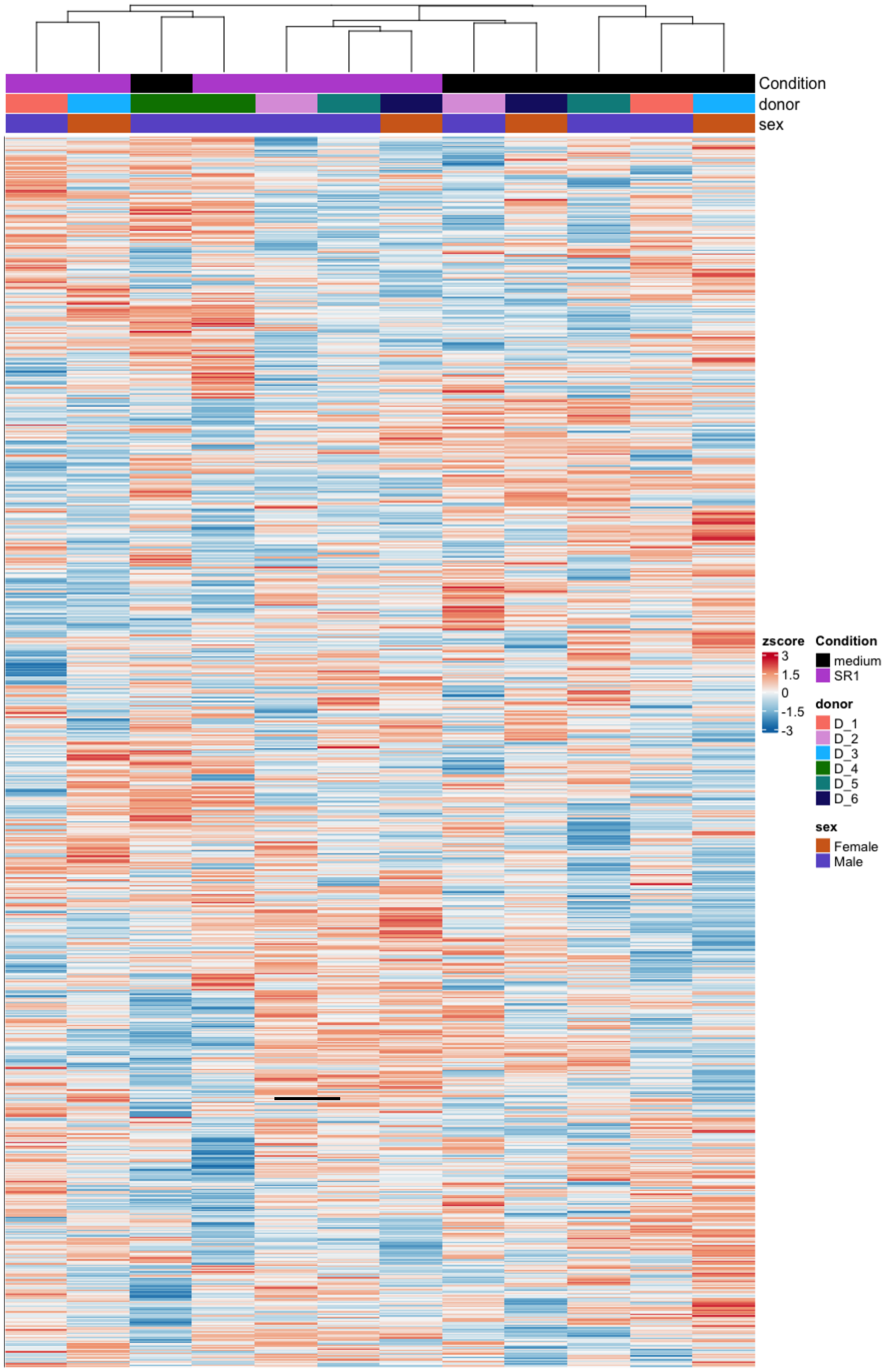

Supplementary figure 5. Endogenous retrovirus expression in human monocytes exposed to AhR antagonist. Human blood monocytes were cultured for 6h in the presence or absence of AhR antagonist SR1. Monocytes were subjected to RNA-seq analysis (n=5). Heatmap of all long terminal repeat elements detected in the transcriptomic dataset. Transcripts names are omitted for readability (see Table S3).

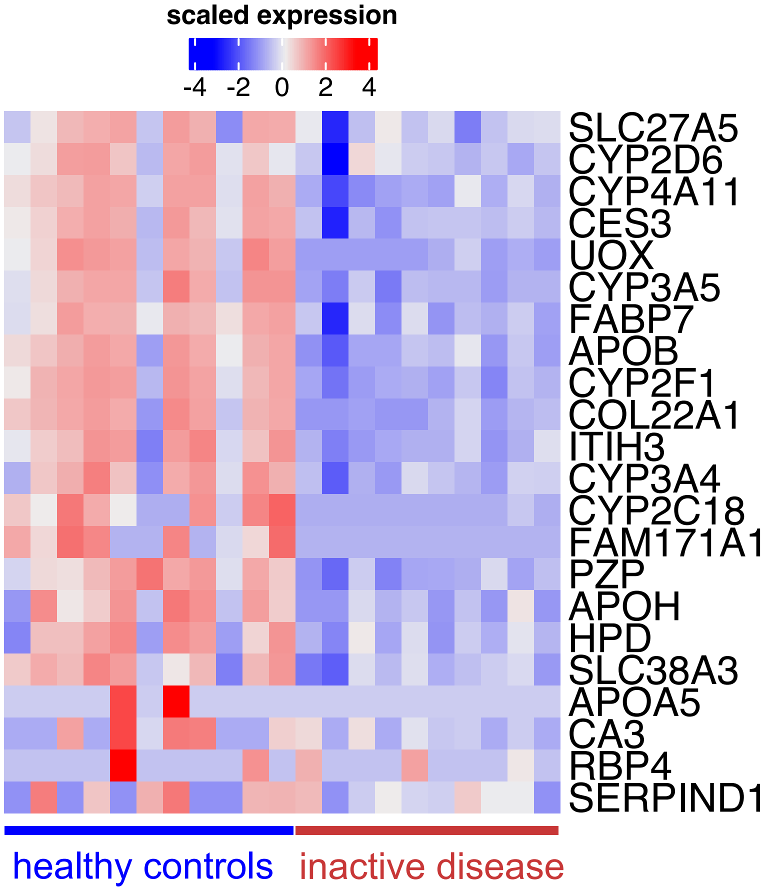

Supplementary figure 6. Transcriptomic analysis of blood monocytes from sJIA patients with inactive disease. Transcriptomic data of blood monocytes from systemic juvenile idiopathic arthritis patients was extracted from public source (GSE147608). Scaled expression of selected genes in healthy controls compared to patients with inactive disease.

Supplementary tables

Table S1. List of differentially expressed genes between WT and AhR-deficient mouse bone marrow monocytes. Genes with log2FoldChange>0 are enriched in KO samples, and genes with log2FoldChange<0 are enriched in WT samples.

Table S2. List of differentially expressed genes between human blood monocytes exposed to AhR antagonist versus medium condition. Genes with log2FoldChange>0 are enriched in 'medium' condition, and genes with log2FoldChange<0 are enriched in SR1 condition.

Table S3. List of differentially expressed long terminal repeat elements between human blood monocytes exposed to AhR antagonist versus medium condition. Genes with log2FoldChange>0 are enriched in SR1 condition, and genes with log2FoldChange<0 are enriched in 'medium' condition.

Table S4. List of genes included in gene signatures used for GSEA. For each indicated signature, genes and their contribution to enrichment are listed.
